# Supplementary material for: Recurrent activating mutations of PPARγ associated with luminal bladder tumors
Source: Nat Commun. 2019 Jan 16;10:253. doi: 10.1038/s41467-018-08157-y (PMC6335423; doi:10.1038/s41467-018-08157-y)
Supplement: Supplementary file 1 — Supplementary Information [file 41467_2018_8157_MOESM1_ESM.pptx]

## Slide 1
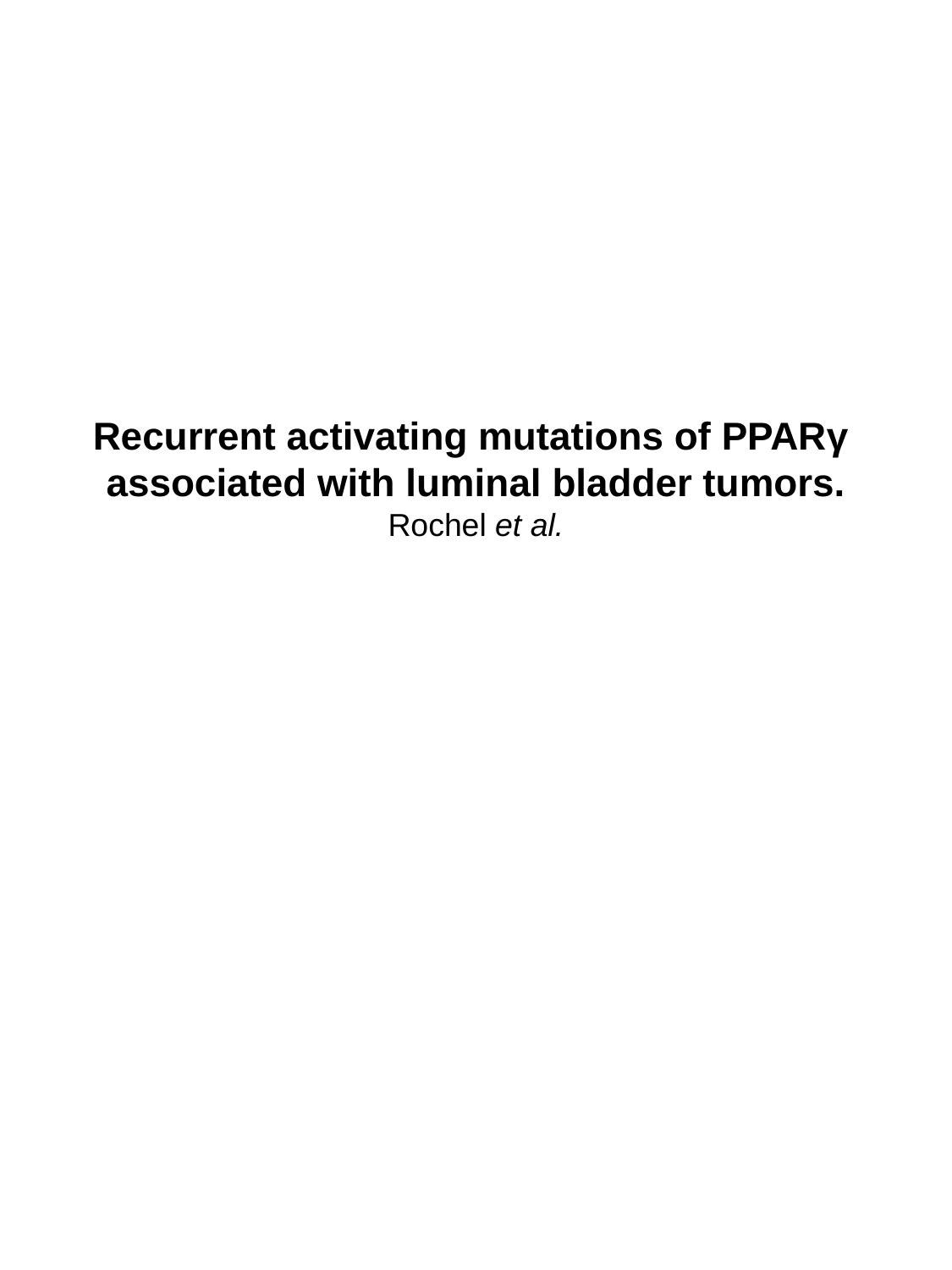

Recurrent activating mutations of PPARγ associated with luminal bladder tumors.
Rochel et al.

## Slide 2
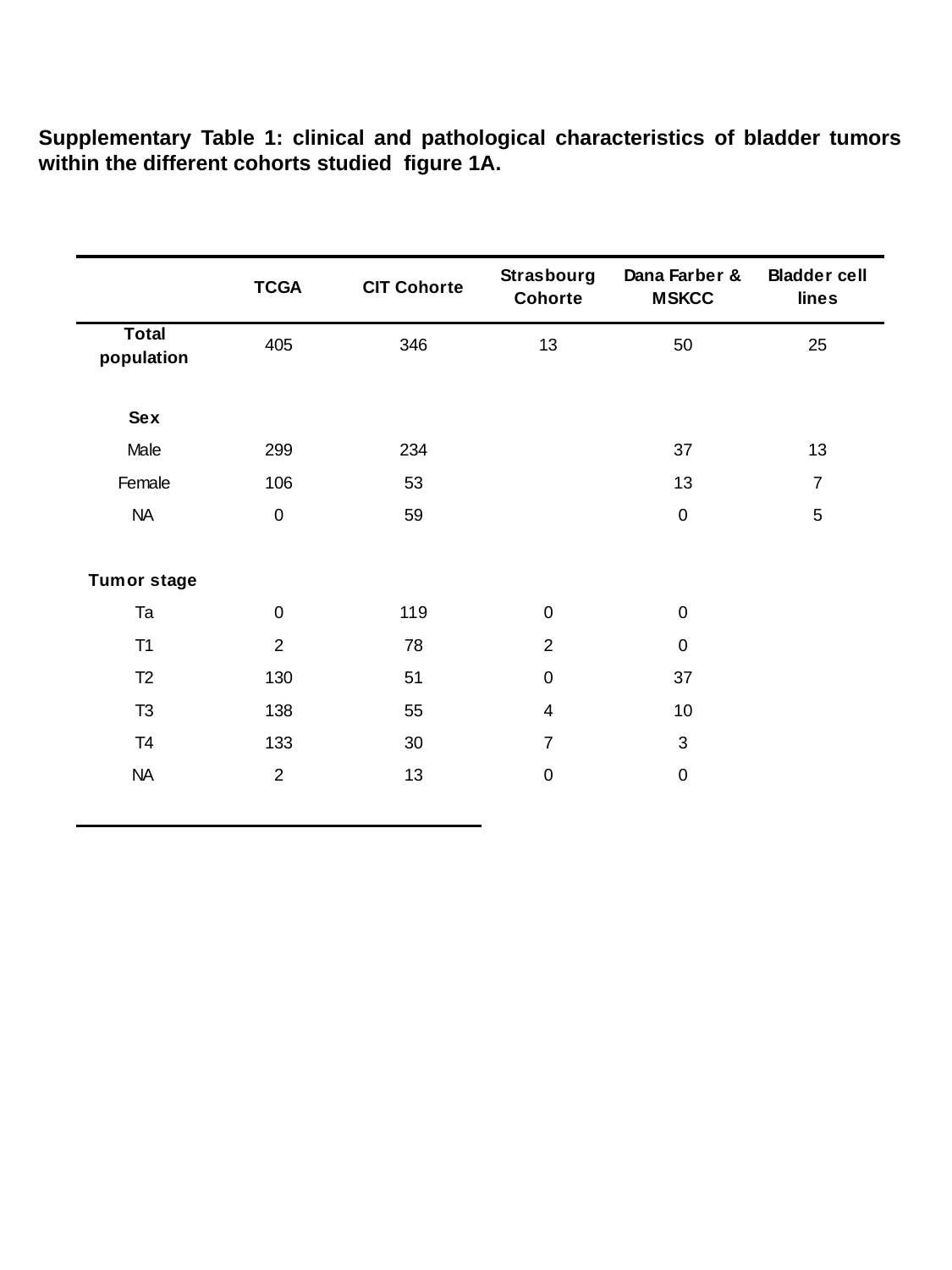

Supplementary Table 1: clinical and pathological characteristics of bladder tumors within the different cohorts studied figure 1A.

## Slide 3
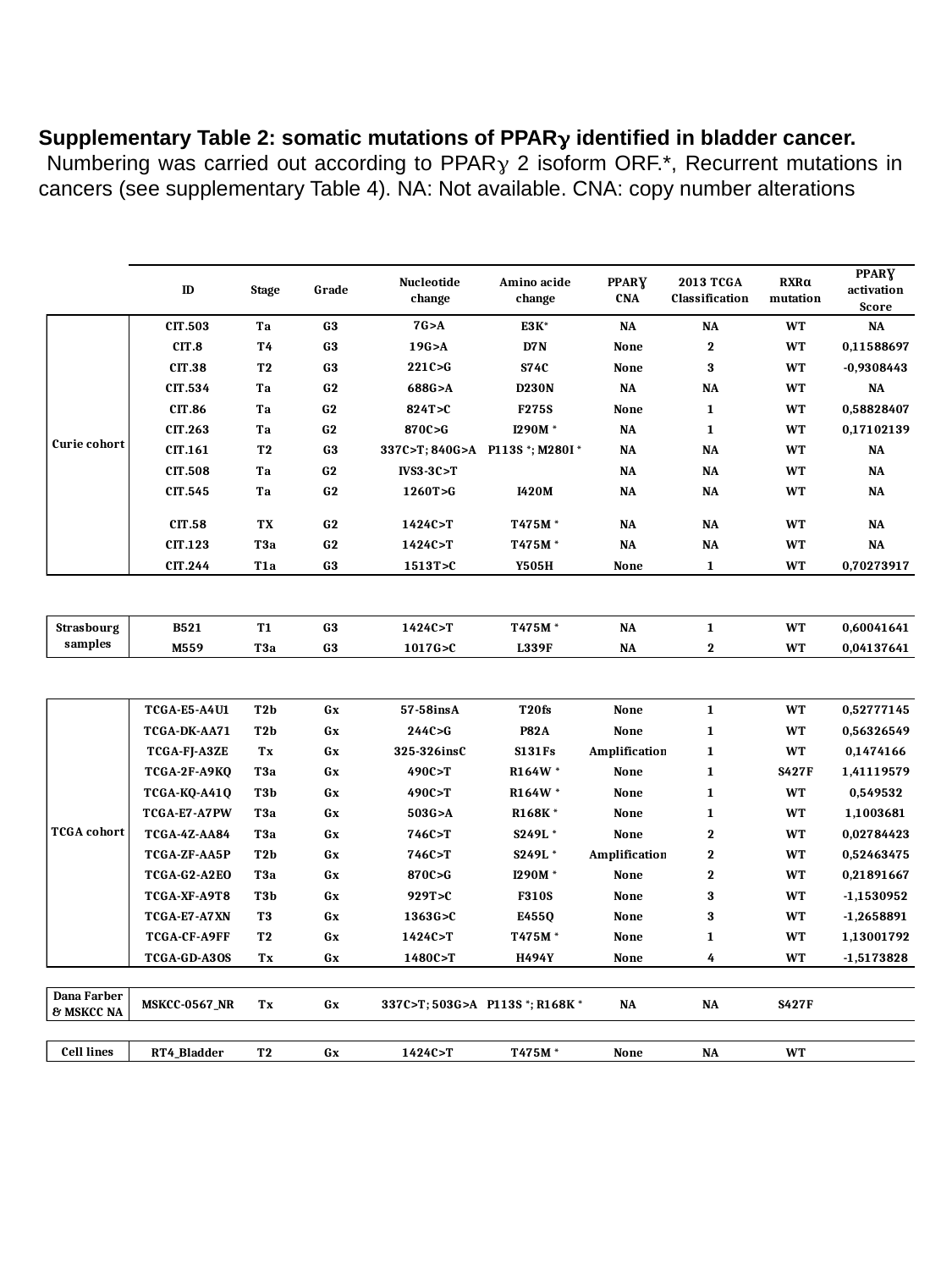

Supplementary Table 2: somatic mutations of PPARg identified in bladder cancer.
 Numbering was carried out according to PPARg 2 isoform ORF.*, Recurrent mutations in cancers (see supplementary Table 4). NA: Not available. CNA: copy number alterations

## Slide 4
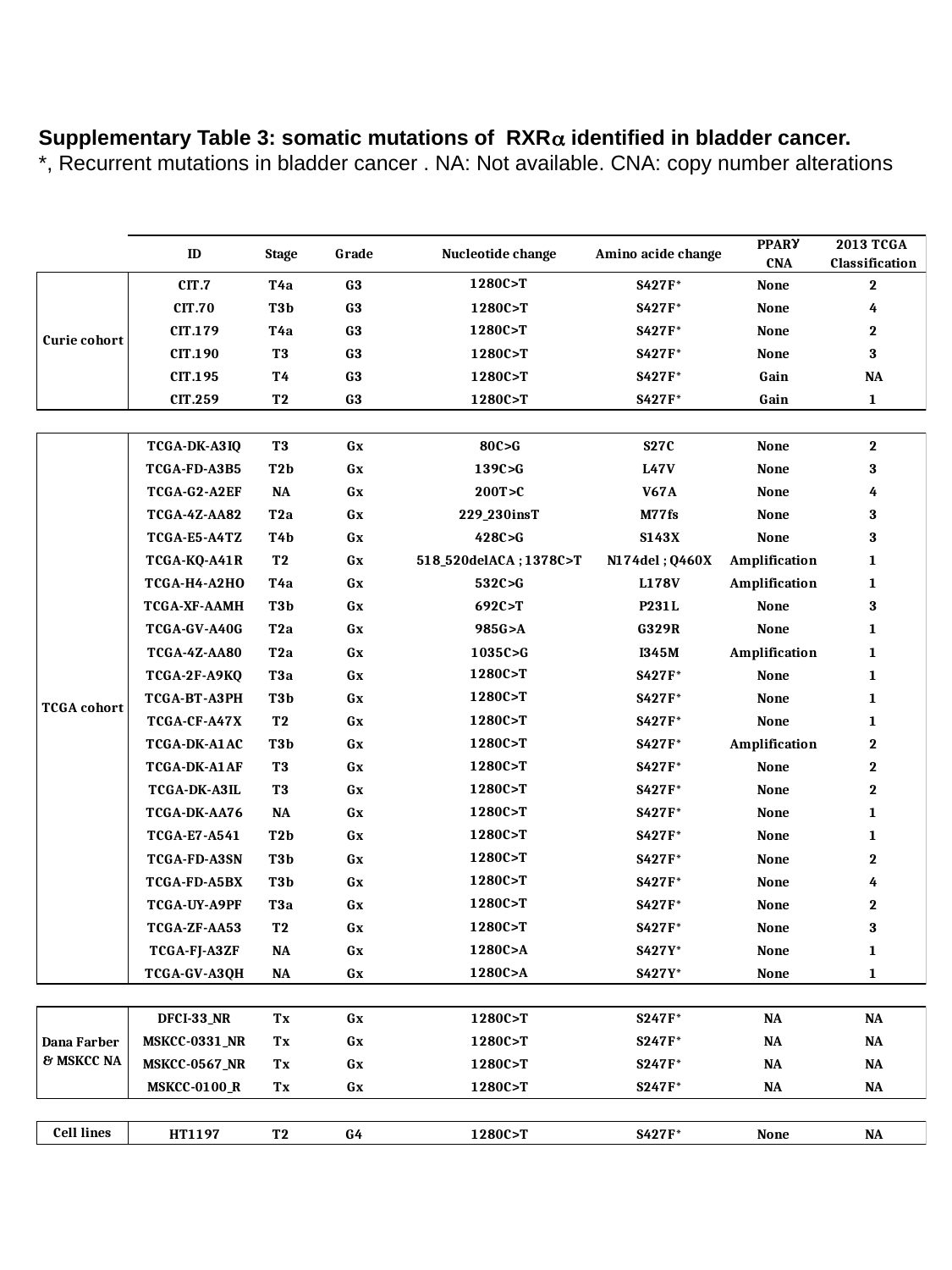

Supplementary Table 3: somatic mutations of RXRa identified in bladder cancer.
*, Recurrent mutations in bladder cancer . NA: Not available. CNA: copy number alterations

## Slide 5
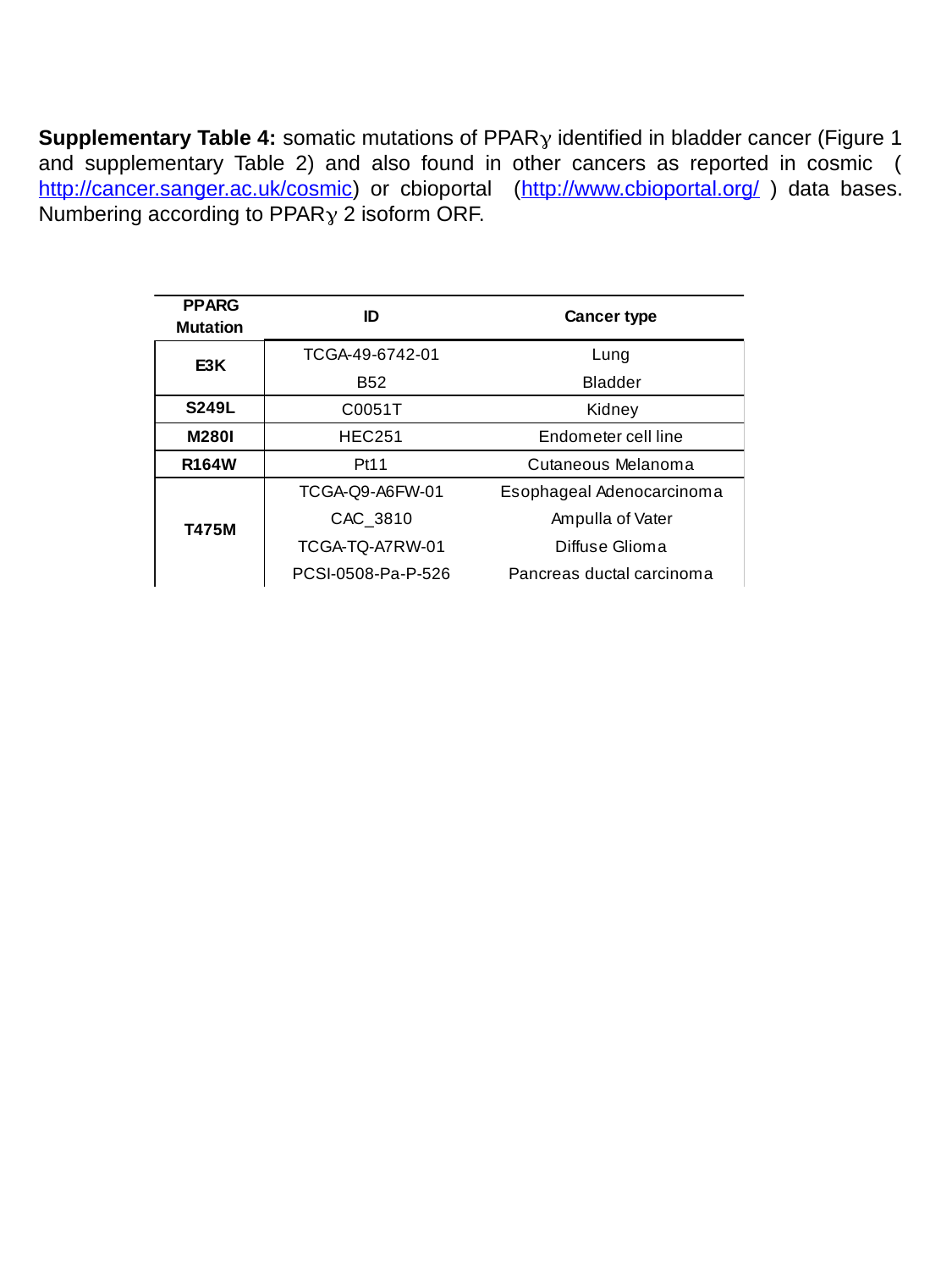

Supplementary Table 4: somatic mutations of PPARg identified in bladder cancer (Figure 1 and supplementary Table 2) and also found in other cancers as reported in cosmic (http://cancer.sanger.ac.uk/cosmic) or cbioportal (http://www.cbioportal.org/ ) data bases. Numbering according to PPARg 2 isoform ORF.

## Slide 6
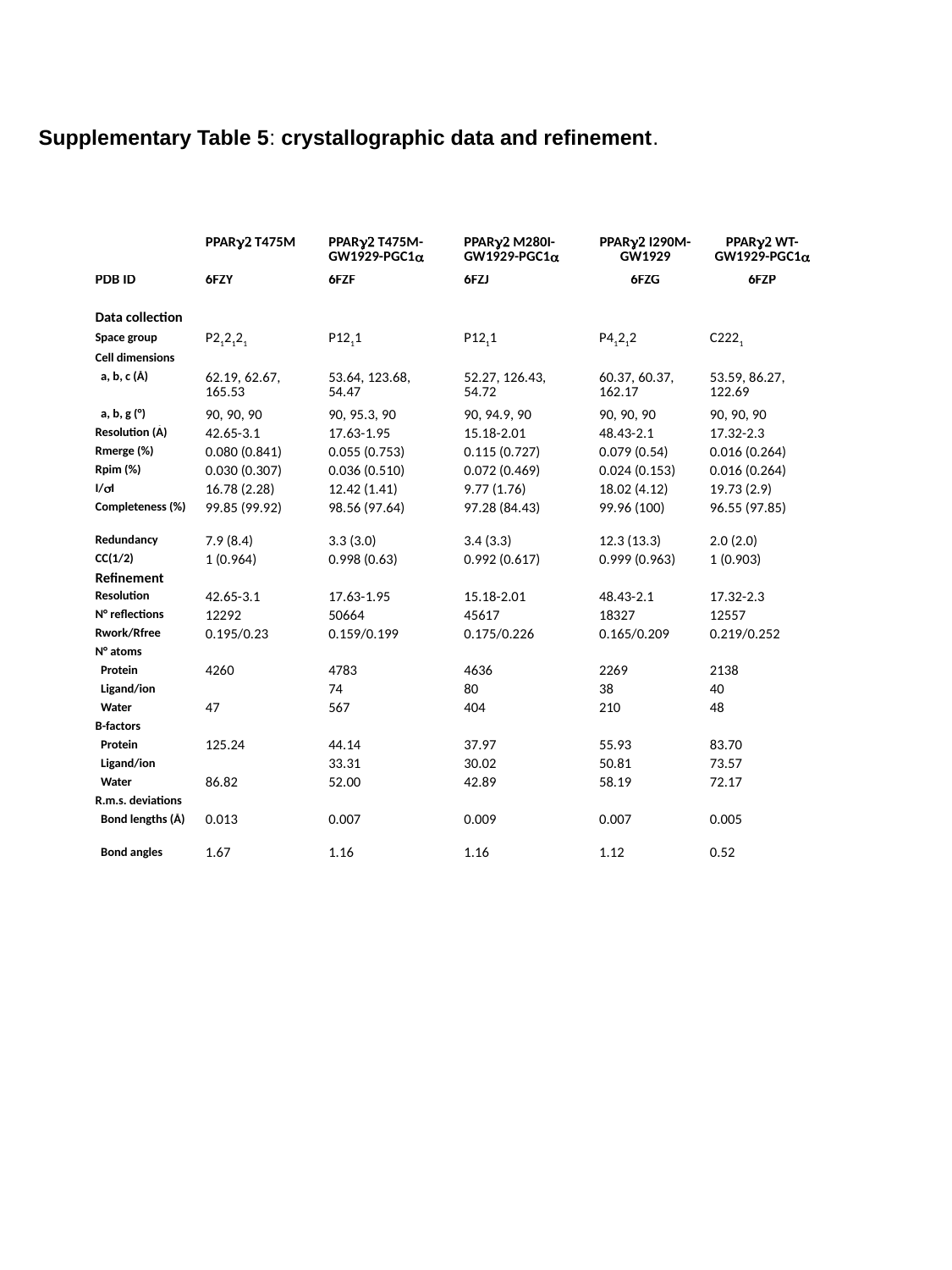

Supplementary Table 5: crystallographic data and refinement.
| | PPARg2 T475M | PPARg2 T475M-GW1929-PGC1a | PPARg2 M280I-GW1929-PGC1a | PPARg2 I290M-GW1929 | PPARg2 WT-GW1929-PGC1a |
| --- | --- | --- | --- | --- | --- |
| PDB ID | 6FZY | 6FZF | 6FZJ | 6FZG | 6FZP |
| Data collection | | | | | |
| Space group | P212121 | P1211 | P1211 | P41212 | C2221 |
| Cell dimensions | | | | | |
| a, b, c (Å) | 62.19, 62.67, 165.53 | 53.64, 123.68, 54.47 | 52.27, 126.43, 54.72 | 60.37, 60.37, 162.17 | 53.59, 86.27, 122.69 |
| a, b, g (°) | 90, 90, 90 | 90, 95.3, 90 | 90, 94.9, 90 | 90, 90, 90 | 90, 90, 90 |
| Resolution (Å) | 42.65-3.1 | 17.63-1.95 | 15.18-2.01 | 48.43-2.1 | 17.32-2.3 |
| Rmerge (%) | 0.080 (0.841) | 0.055 (0.753) | 0.115 (0.727) | 0.079 (0.54) | 0.016 (0.264) |
| Rpim (%) | 0.030 (0.307) | 0.036 (0.510) | 0.072 (0.469) | 0.024 (0.153) | 0.016 (0.264) |
| I/sI | 16.78 (2.28) | 12.42 (1.41) | 9.77 (1.76) | 18.02 (4.12) | 19.73 (2.9) |
| Completeness (%) | 99.85 (99.92) | 98.56 (97.64) | 97.28 (84.43) | 99.96 (100) | 96.55 (97.85) |
| Redundancy | 7.9 (8.4) | 3.3 (3.0) | 3.4 (3.3) | 12.3 (13.3) | 2.0 (2.0) |
| CC(1/2) | 1 (0.964) | 0.998 (0.63) | 0.992 (0.617) | 0.999 (0.963) | 1 (0.903) |
| Refinement | | | | | |
| Resolution | 42.65-3.1 | 17.63-1.95 | 15.18-2.01 | 48.43-2.1 | 17.32-2.3 |
| N° reflections | 12292 | 50664 | 45617 | 18327 | 12557 |
| Rwork/Rfree | 0.195/0.23 | 0.159/0.199 | 0.175/0.226 | 0.165/0.209 | 0.219/0.252 |
| N° atoms | | | | | |
| Protein | 4260 | 4783 | 4636 | 2269 | 2138 |
| Ligand/ion | | 74 | 80 | 38 | 40 |
| Water | 47 | 567 | 404 | 210 | 48 |
| B-factors | | | | | |
| Protein | 125.24 | 44.14 | 37.97 | 55.93 | 83.70 |
| Ligand/ion | | 33.31 | 30.02 | 50.81 | 73.57 |
| Water | 86.82 | 52.00 | 42.89 | 58.19 | 72.17 |
| R.m.s. deviations | | | | | |
| Bond lengths (Å) | 0.013 | 0.007 | 0.009 | 0.007 | 0.005 |
| Bond angles | 1.67 | 1.16 | 1.16 | 1.12 | 0.52 |

## Slide 7
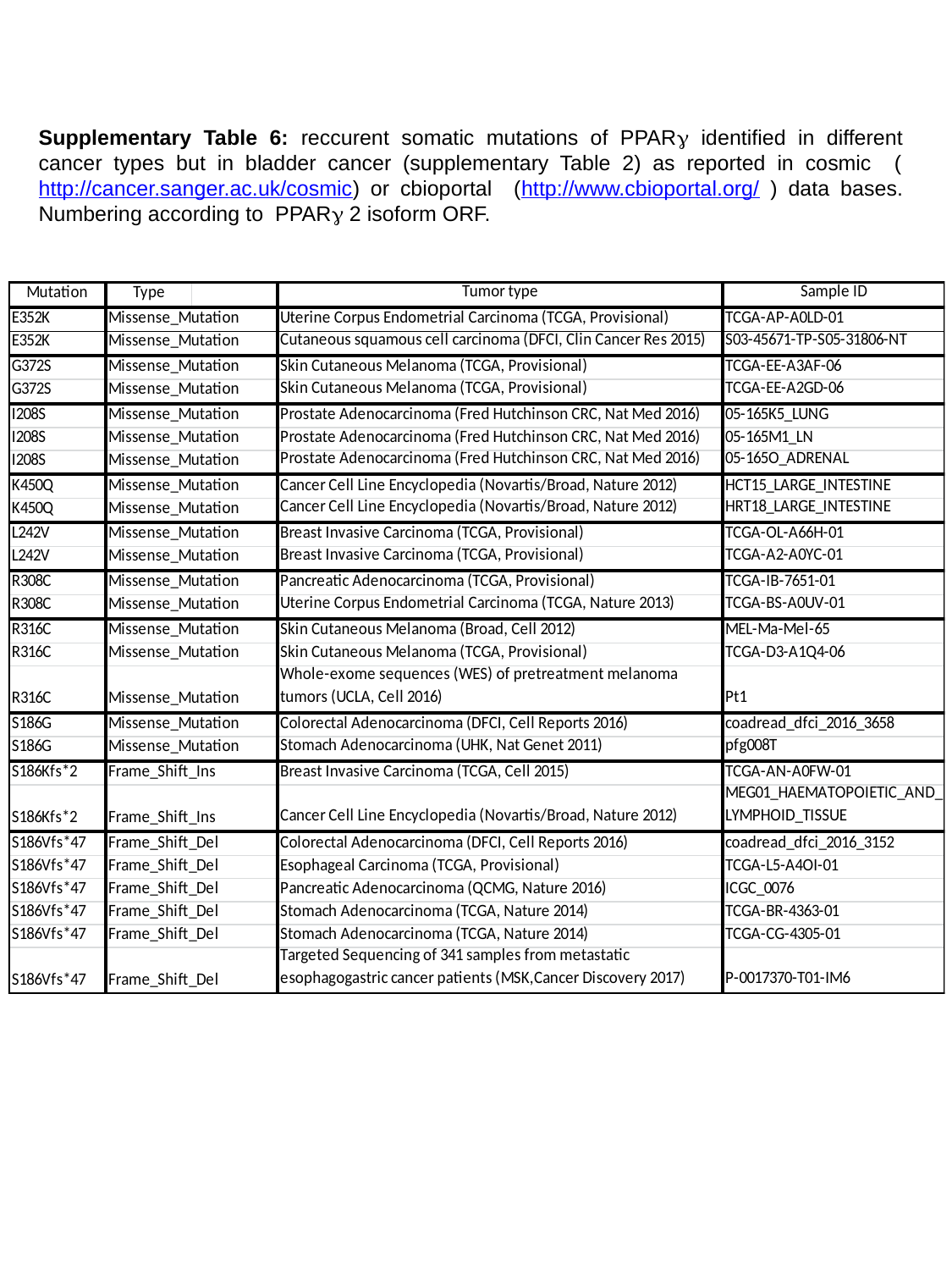

Supplementary Table 6: reccurent somatic mutations of PPARg identified in different cancer types but in bladder cancer (supplementary Table 2) as reported in cosmic (http://cancer.sanger.ac.uk/cosmic) or cbioportal (http://www.cbioportal.org/ ) data bases. Numbering according to PPARg 2 isoform ORF.

## Slide 8
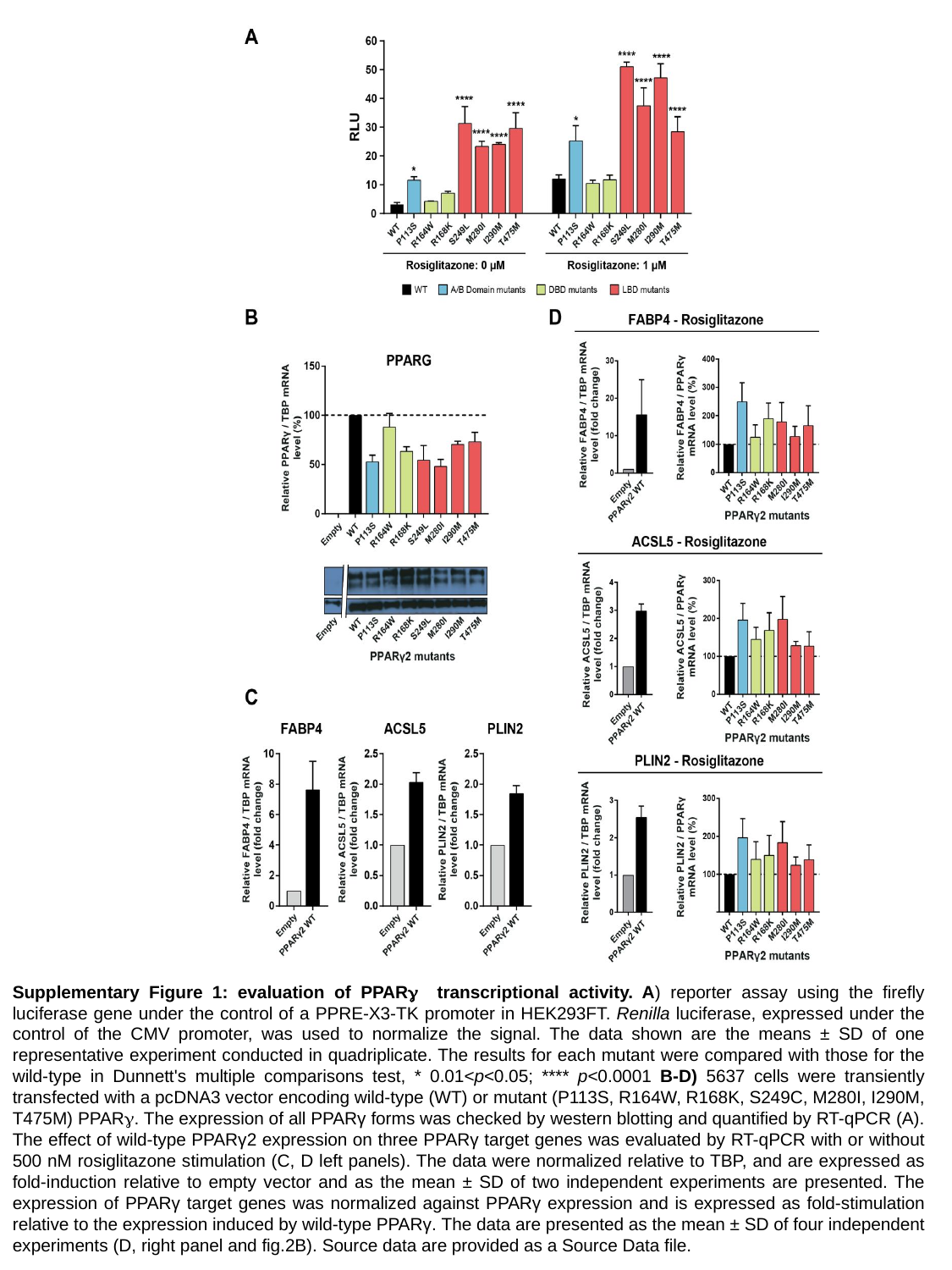

Supplementary Figure 1: evaluation of PPARg transcriptional activity. A) reporter assay using the firefly luciferase gene under the control of a PPRE-X3-TK promoter in HEK293FT. Renilla luciferase, expressed under the control of the CMV promoter, was used to normalize the signal. The data shown are the means ± SD of one representative experiment conducted in quadriplicate. The results for each mutant were compared with those for the wild-type in Dunnett's multiple comparisons test, * 0.01<p<0.05; **** p<0.0001 B-D) 5637 cells were transiently transfected with a pcDNA3 vector encoding wild-type (WT) or mutant (P113S, R164W, R168K, S249C, M280I, I290M, T475M) PPARg. The expression of all PPARγ forms was checked by western blotting and quantified by RT-qPCR (A). The effect of wild-type PPARγ2 expression on three PPARγ target genes was evaluated by RT-qPCR with or without 500 nM rosiglitazone stimulation (C, D left panels). The data were normalized relative to TBP, and are expressed as fold-induction relative to empty vector and as the mean ± SD of two independent experiments are presented. The expression of PPARγ target genes was normalized against PPARγ expression and is expressed as fold-stimulation relative to the expression induced by wild-type PPARγ. The data are presented as the mean ± SD of four independent experiments (D, right panel and fig.2B). Source data are provided as a Source Data file.

## Slide 9
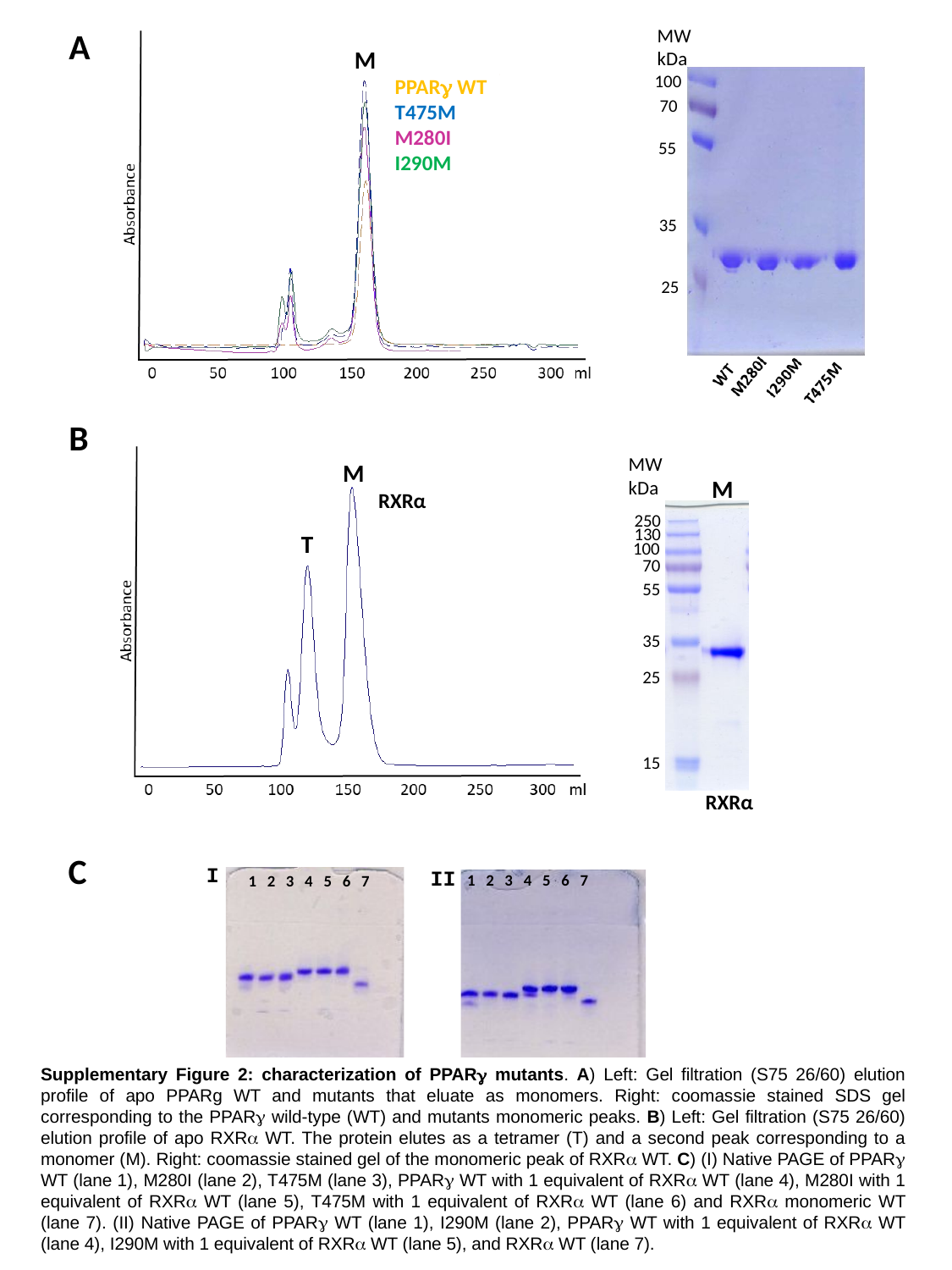

MW
kDa
100
70
55
35
25
A
M
PPARg WT
T475M
M280I
I290M
B
MW
kDa
250
130
100
70
55
35
25
15
M
M
RXRα
T
RXRα
C
I
II
1 2 3 4 5 6 7
1 2 3 4 5 6 7
Supplementary Figure 2: characterization of PPARg mutants. A) Left: Gel filtration (S75 26/60) elution profile of apo PPARg WT and mutants that eluate as monomers. Right: coomassie stained SDS gel corresponding to the PPARg wild-type (WT) and mutants monomeric peaks. B) Left: Gel filtration (S75 26/60) elution profile of apo RXRa WT. The protein elutes as a tetramer (T) and a second peak corresponding to a monomer (M). Right: coomassie stained gel of the monomeric peak of RXRa WT. C) (I) Native PAGE of PPARg WT (lane 1), M280I (lane 2), T475M (lane 3), PPARg WT with 1 equivalent of RXRa WT (lane 4), M280I with 1 equivalent of RXRa WT (lane 5), T475M with 1 equivalent of RXRa WT (lane 6) and RXRa monomeric WT (lane 7). (II) Native PAGE of PPARg WT (lane 1), I290M (lane 2), PPARg WT with 1 equivalent of RXRa WT (lane 4), I290M with 1 equivalent of RXRa WT (lane 5), and RXRa WT (lane 7).

## Slide 10
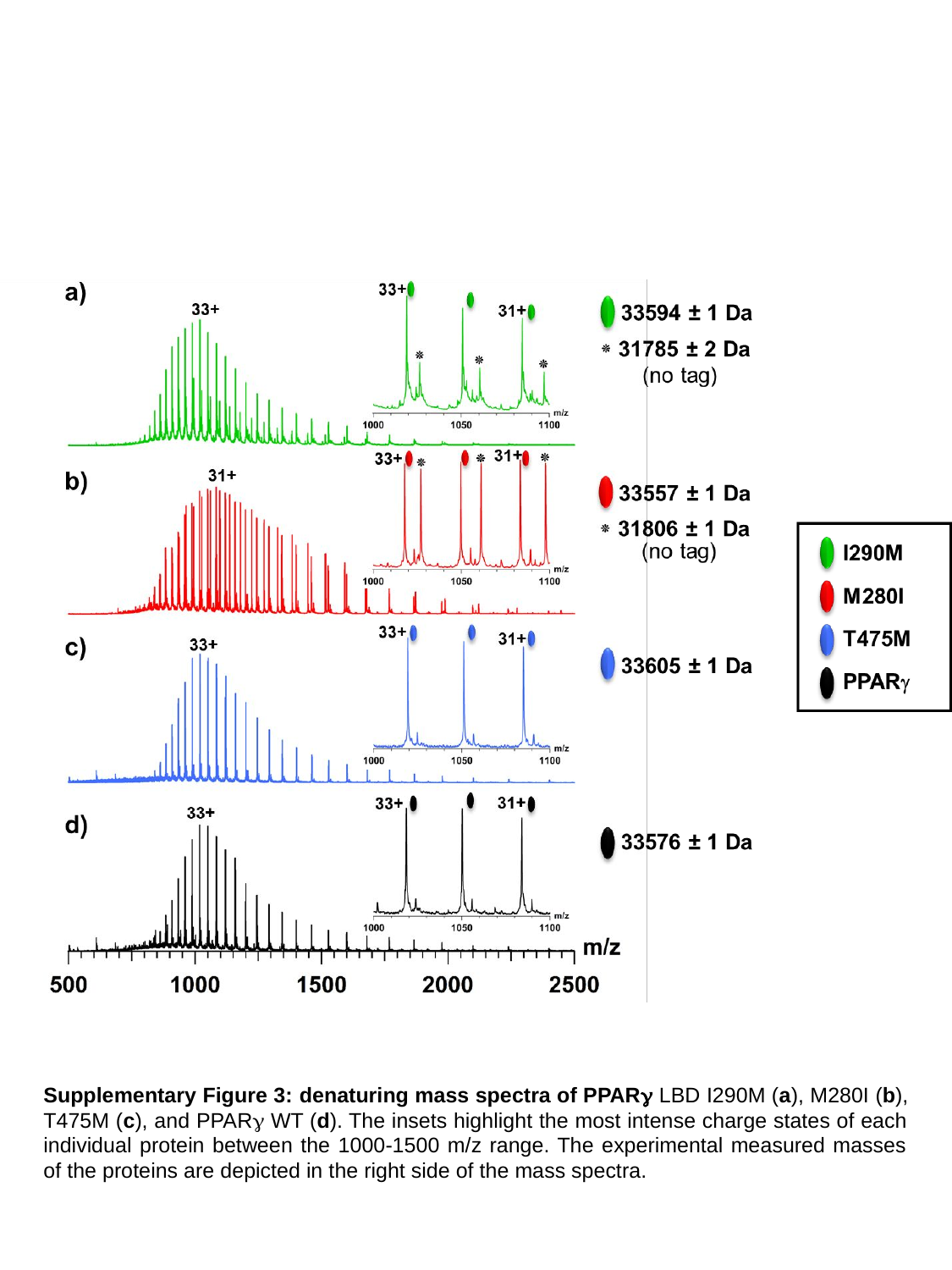

Supplementary Figure 3: denaturing mass spectra of PPARγ LBD I290M (a), M280I (b), T475M (c), and PPARg WT (d). The insets highlight the most intense charge states of each individual protein between the 1000-1500 m/z range. The experimental measured masses of the proteins are depicted in the right side of the mass spectra.

## Slide 11
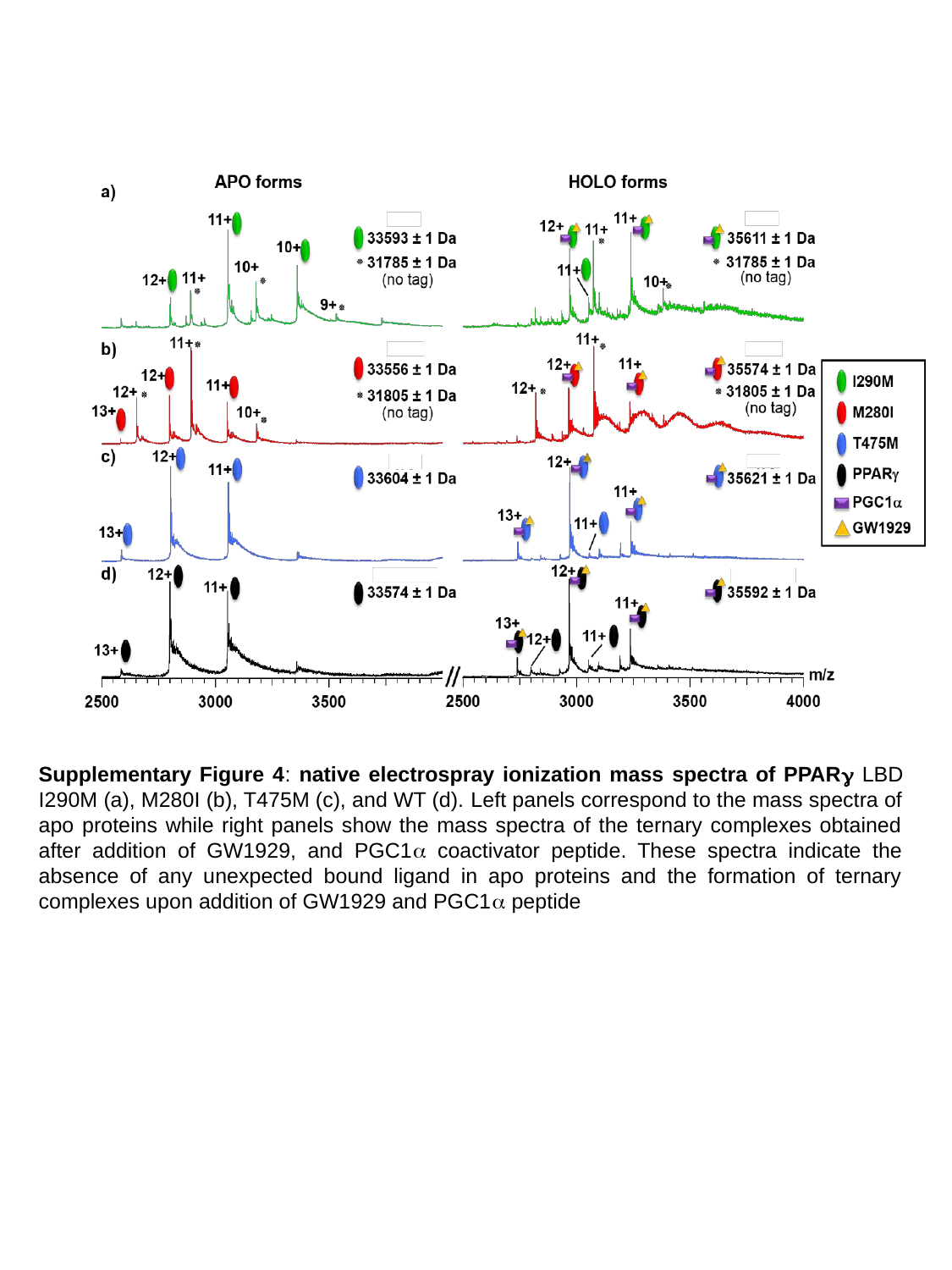

Supplementary Figure 4: native electrospray ionization mass spectra of PPARg LBD I290M (a), M280I (b), T475M (c), and WT (d). Left panels correspond to the mass spectra of apo proteins while right panels show the mass spectra of the ternary complexes obtained after addition of GW1929, and PGC1a coactivator peptide. These spectra indicate the absence of any unexpected bound ligand in apo proteins and the formation of ternary complexes upon addition of GW1929 and PGC1a peptide

## Slide 12
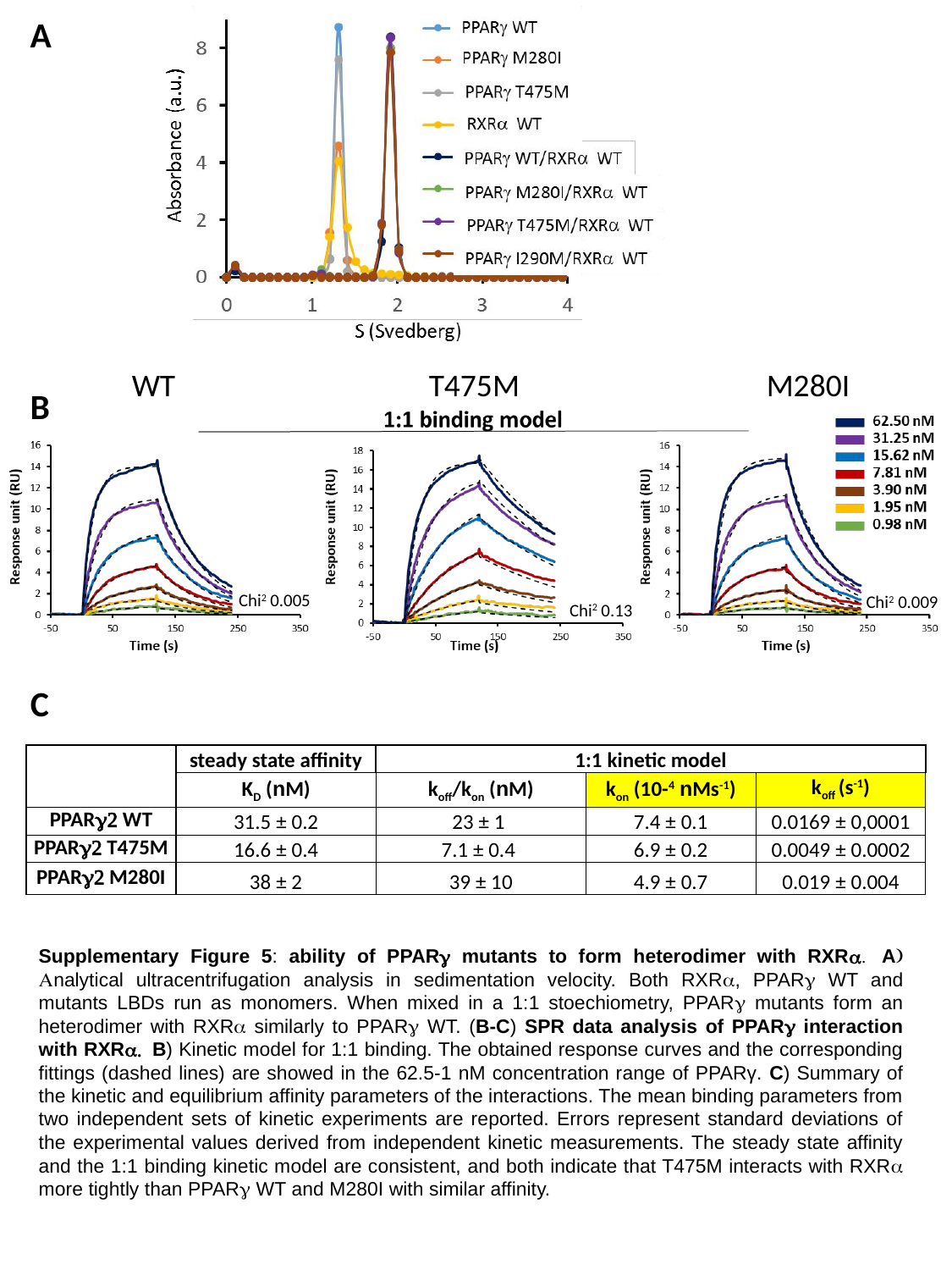

A
WT		 T475M		M280I
B
Chi2 0.005
Chi2 0.009
Chi2 0.13
C
| | steady state affinity | 1:1 kinetic model | | |
| --- | --- | --- | --- | --- |
| | KD (nM) | koff/kon (nM) | kon (10-4 nMs-1) | koff (s-1) |
| PPARg2 WT | 31.5 ± 0.2 | 23 ± 1 | 7.4 ± 0.1 | 0.0169 ± 0,0001 |
| PPARg2 T475M | 16.6 ± 0.4 | 7.1 ± 0.4 | 6.9 ± 0.2 | 0.0049 ± 0.0002 |
| PPARg2 M280I | 38 ± 2 | 39 ± 10 | 4.9 ± 0.7 | 0.019 ± 0.004 |
Supplementary Figure 5: ability of PPARg mutants to form heterodimer with RXRa. A) Analytical ultracentrifugation analysis in sedimentation velocity. Both RXRa, PPARg WT and mutants LBDs run as monomers. When mixed in a 1:1 stoechiometry, PPARg mutants form an heterodimer with RXRa similarly to PPARg WT. (B-C) SPR data analysis of PPARg interaction with RXRa. B) Kinetic model for 1:1 binding. The obtained response curves and the corresponding fittings (dashed lines) are showed in the 62.5-1 nM concentration range of PPARγ. C) Summary of the kinetic and equilibrium affinity parameters of the interactions. The mean binding parameters from two independent sets of kinetic experiments are reported. Errors represent standard deviations of the experimental values derived from independent kinetic measurements. The steady state affinity and the 1:1 binding kinetic model are consistent, and both indicate that T475M interacts with RXRa more tightly than PPARg WT and M280I with similar affinity.

## Slide 13
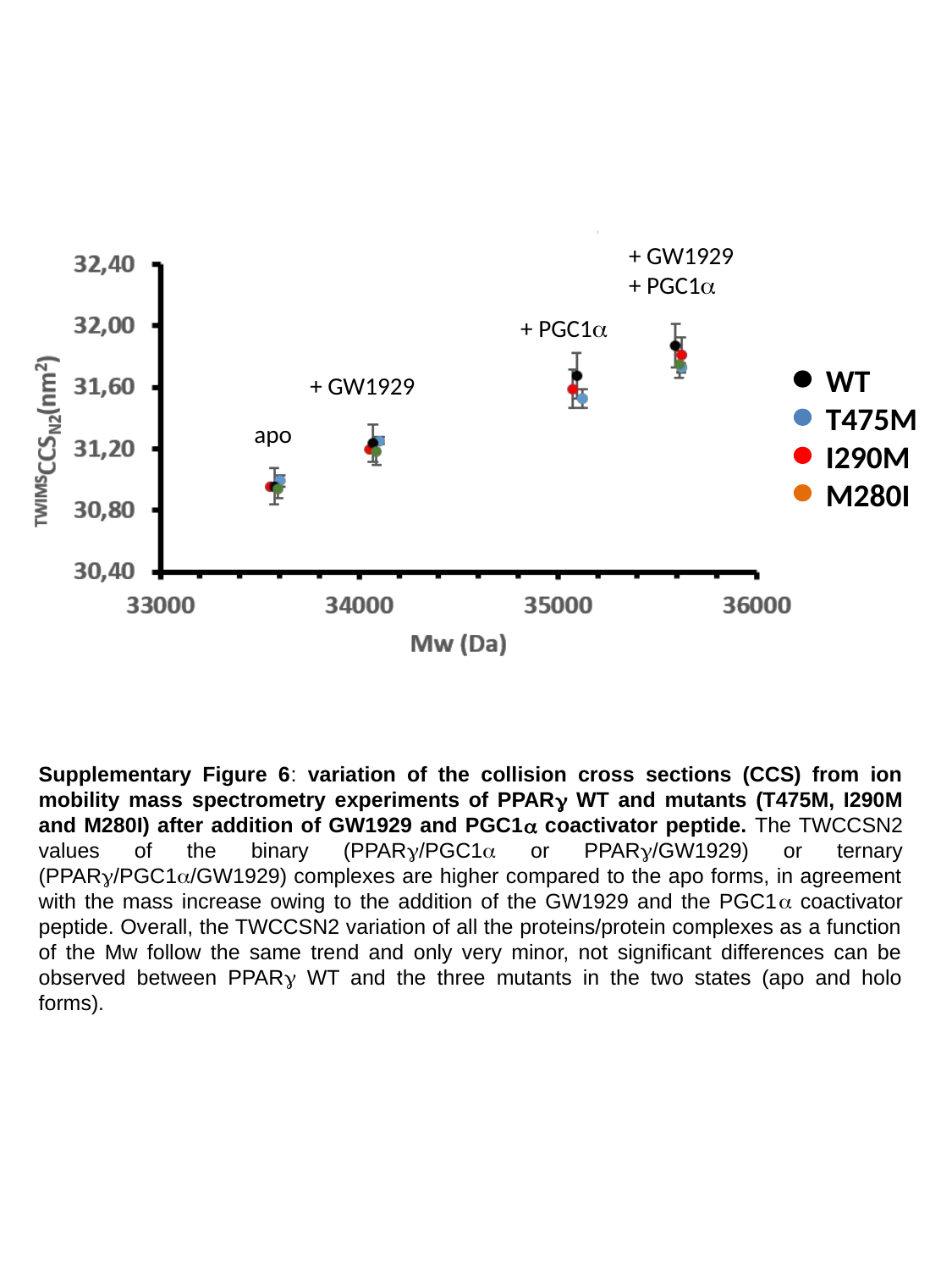

+GW+HP33
wt-PPAR
475
I290
M280
+GW
apo
+ GW1929
+ PGC1a
+ PGC1a
WT
T475M
I290M
M280I
+ GW1929
Supplementary Figure 6: variation of the collision cross sections (CCS) from ion mobility mass spectrometry experiments of PPARg WT and mutants (T475M, I290M and M280I) after addition of GW1929 and PGC1a coactivator peptide. The TWCCSN2 values of the binary (PPARg/PGC1a or PPARg/GW1929) or ternary (PPARg/PGC1a/GW1929) complexes are higher compared to the apo forms, in agreement with the mass increase owing to the addition of the GW1929 and the PGC1a coactivator peptide. Overall, the TWCCSN2 variation of all the proteins/protein complexes as a function of the Mw follow the same trend and only very minor, not significant differences can be observed between PPARg WT and the three mutants in the two states (apo and holo forms).

## Slide 14
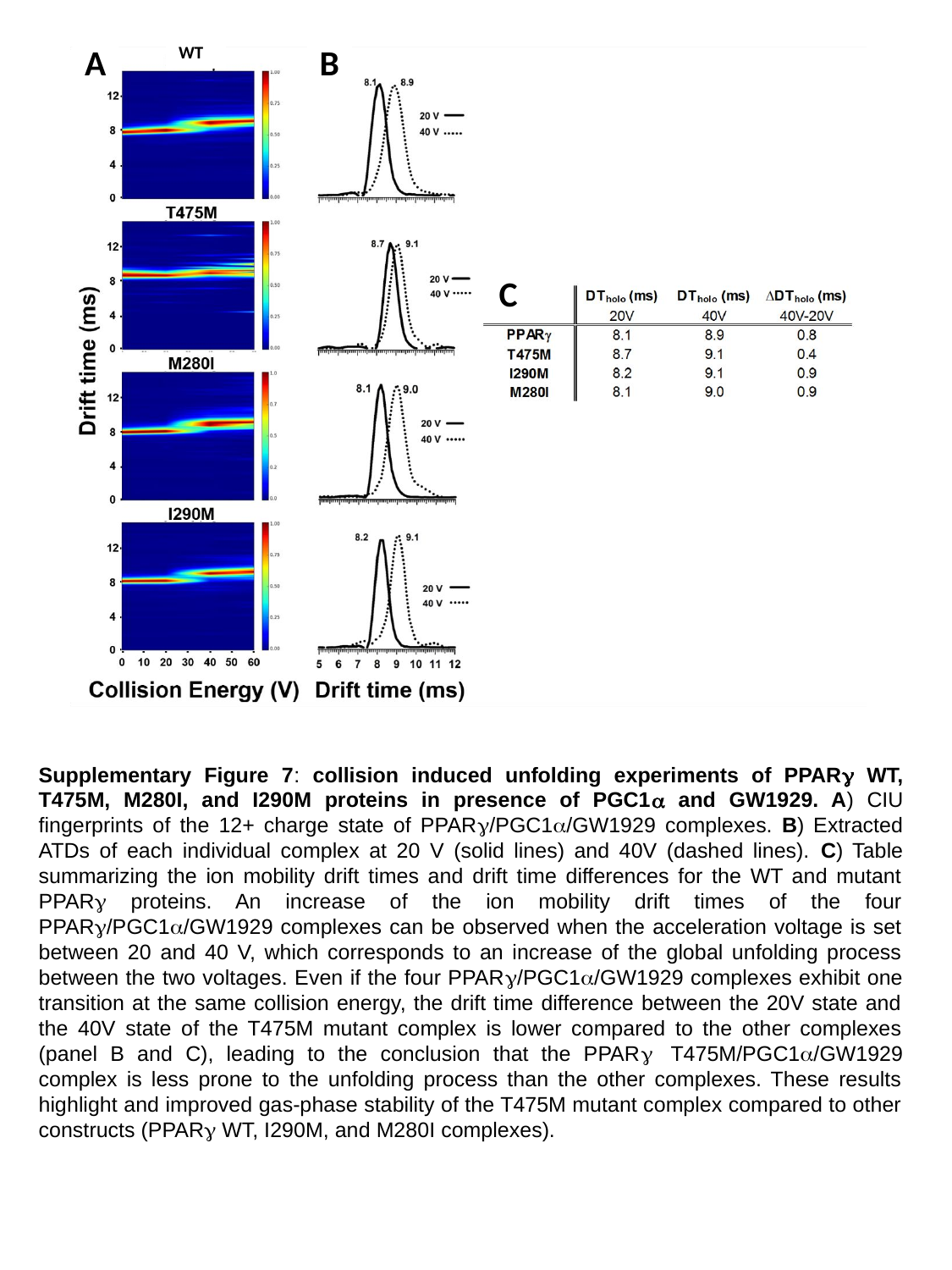

A
B
WT
C
Supplementary Figure 7: collision induced unfolding experiments of PPARγ WT, T475M, M280I, and I290M proteins in presence of PGC1a and GW1929. A) CIU fingerprints of the 12+ charge state of PPARg/PGC1a/GW1929 complexes. B) Extracted ATDs of each individual complex at 20 V (solid lines) and 40V (dashed lines). C) Table summarizing the ion mobility drift times and drift time differences for the WT and mutant PPARg proteins. An increase of the ion mobility drift times of the four PPARg/PGC1a/GW1929 complexes can be observed when the acceleration voltage is set between 20 and 40 V, which corresponds to an increase of the global unfolding process between the two voltages. Even if the four PPARg/PGC1a/GW1929 complexes exhibit one transition at the same collision energy, the drift time difference between the 20V state and the 40V state of the T475M mutant complex is lower compared to the other complexes (panel B and C), leading to the conclusion that the PPARg T475M/PGC1a/GW1929 complex is less prone to the unfolding process than the other complexes. These results highlight and improved gas-phase stability of the T475M mutant complex compared to other constructs (PPARg WT, I290M, and M280I complexes).

## Slide 15
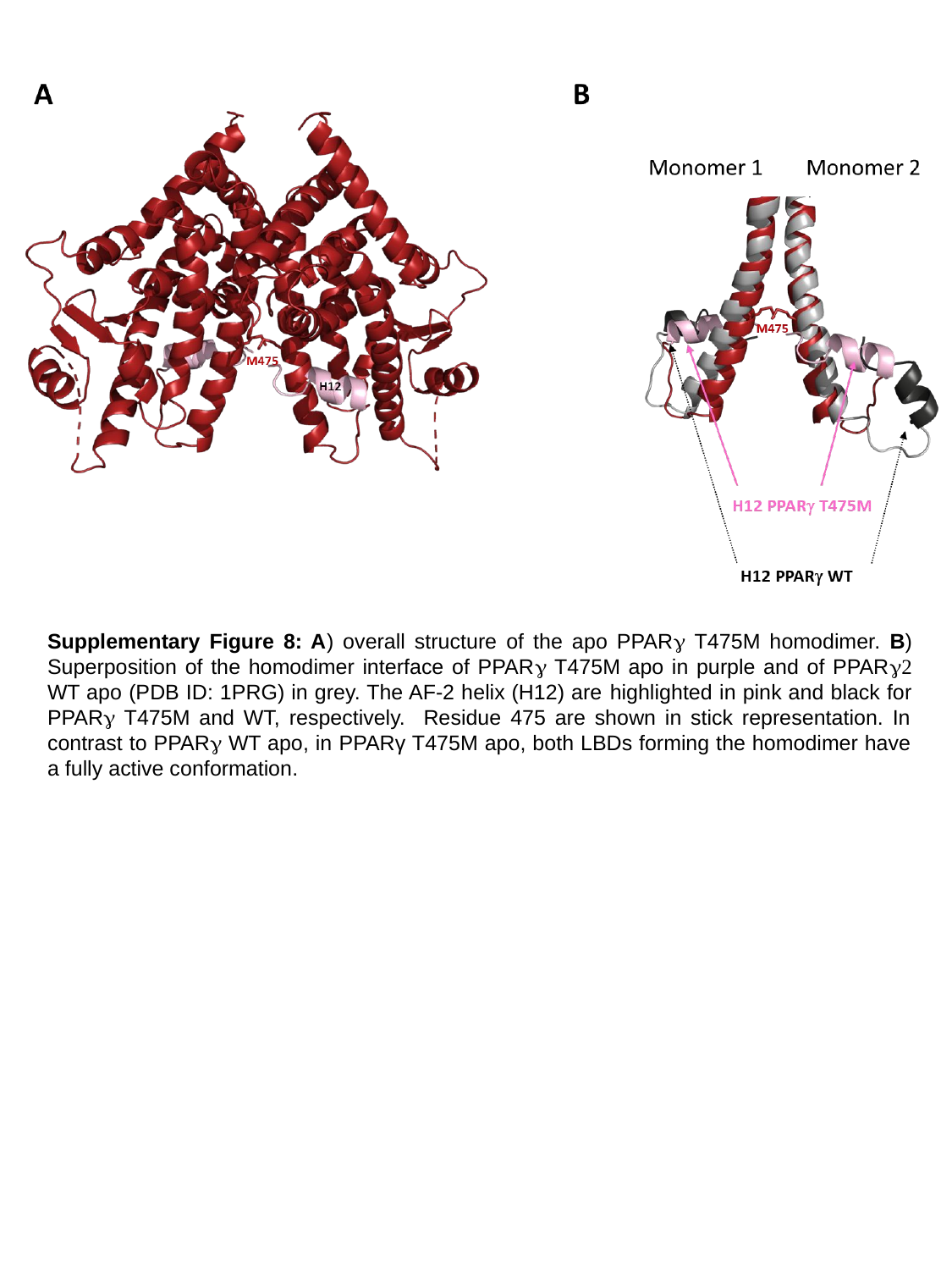

Supplementary Figure 8: A) overall structure of the apo PPARg T475M homodimer. B) Superposition of the homodimer interface of PPARg T475M apo in purple and of PPARg2 WT apo (PDB ID: 1PRG) in grey. The AF-2 helix (H12) are highlighted in pink and black for PPARg T475M and WT, respectively. Residue 475 are shown in stick representation. In contrast to PPARg WT apo, in PPARγ T475M apo, both LBDs forming the homodimer have a fully active conformation.

## Slide 16
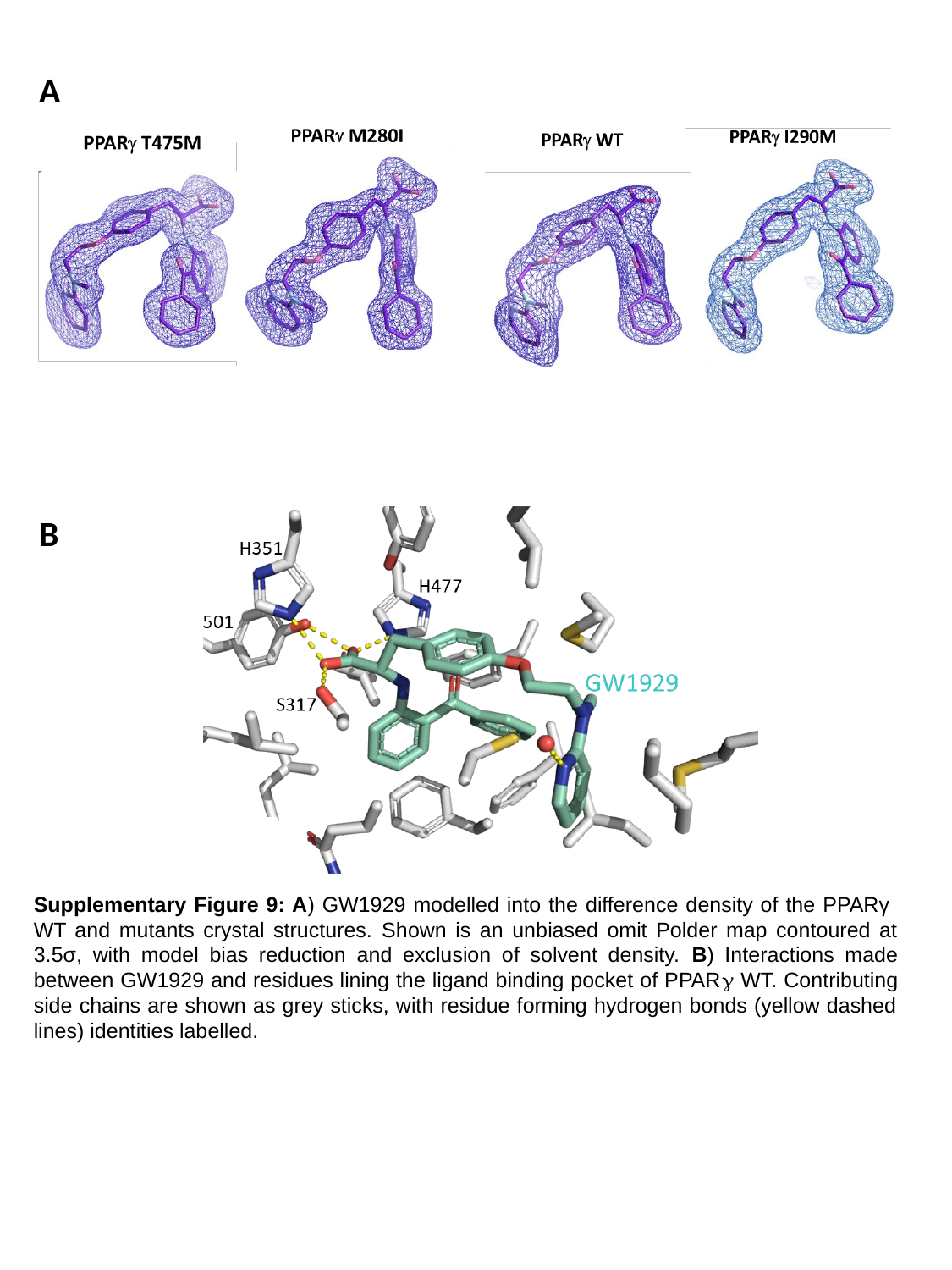

A
B
Supplementary Figure 9: A) GW1929 modelled into the difference density of the PPARγ WT and mutants crystal structures. Shown is an unbiased omit Polder map contoured at 3.5σ, with model bias reduction and exclusion of solvent density. B) Interactions made between GW1929 and residues lining the ligand binding pocket of PPARg WT. Contributing side chains are shown as grey sticks, with residue forming hydrogen bonds (yellow dashed lines) identities labelled.

## Slide 17
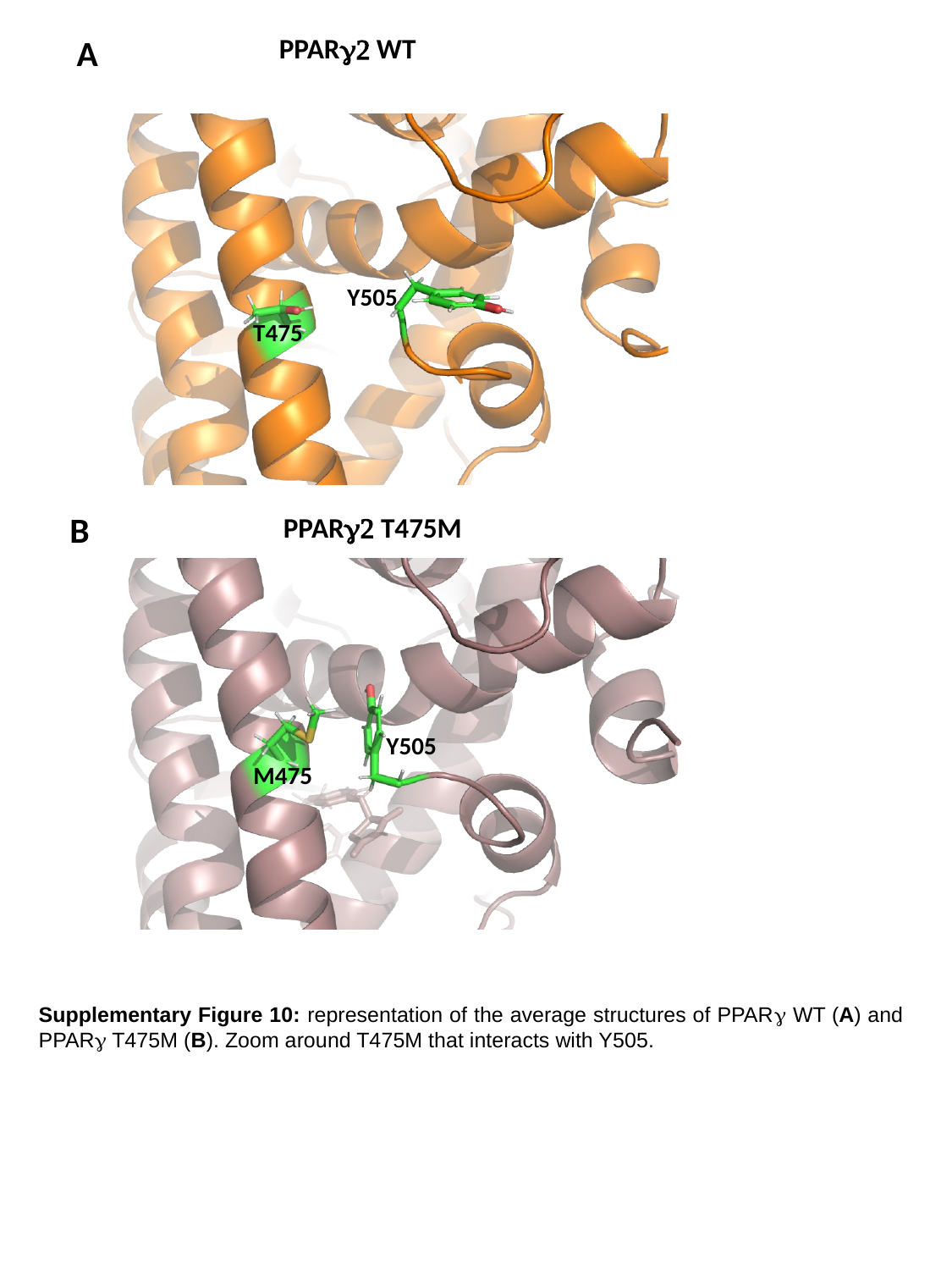

A
PPARg2 WT
Y505
T475
B
PPARg2 T475M
Y505
M475
Supplementary Figure 10: representation of the average structures of PPARg WT (A) and PPARg T475M (B). Zoom around T475M that interacts with Y505.

## Slide 18
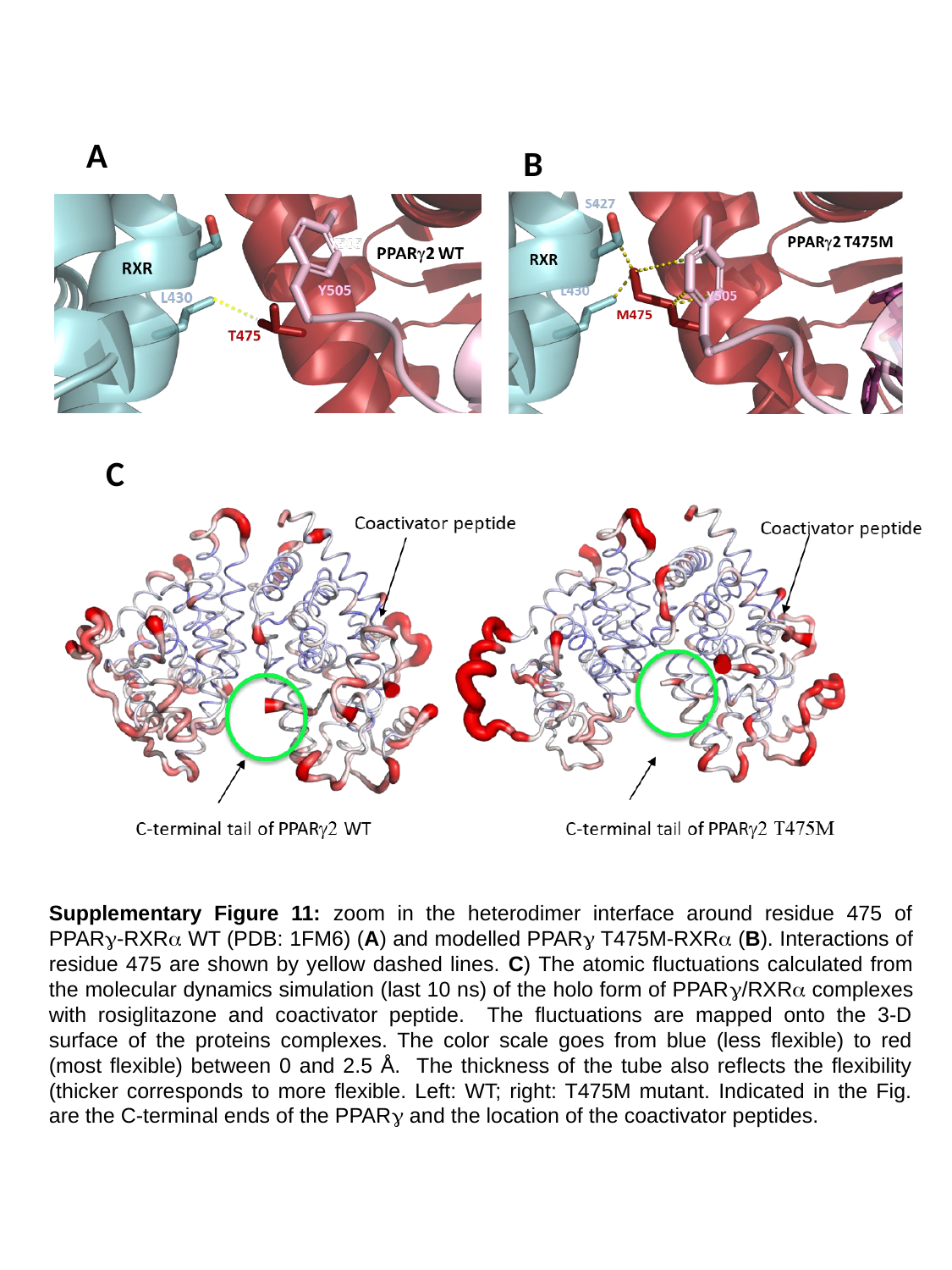

A
B
C
Supplementary Figure 11: zoom in the heterodimer interface around residue 475 of PPARg-RXRa WT (PDB: 1FM6) (A) and modelled PPARg T475M-RXRa (B). Interactions of residue 475 are shown by yellow dashed lines. C) The atomic fluctuations calculated from the molecular dynamics simulation (last 10 ns) of the holo form of PPARg/RXRa complexes with rosiglitazone and coactivator peptide. The fluctuations are mapped onto the 3-D surface of the proteins complexes. The color scale goes from blue (less flexible) to red (most flexible) between 0 and 2.5 Å. The thickness of the tube also reflects the flexibility (thicker corresponds to more flexible. Left: WT; right: T475M mutant. Indicated in the Fig. are the C-terminal ends of the PPARg and the location of the coactivator peptides.

## Slide 19
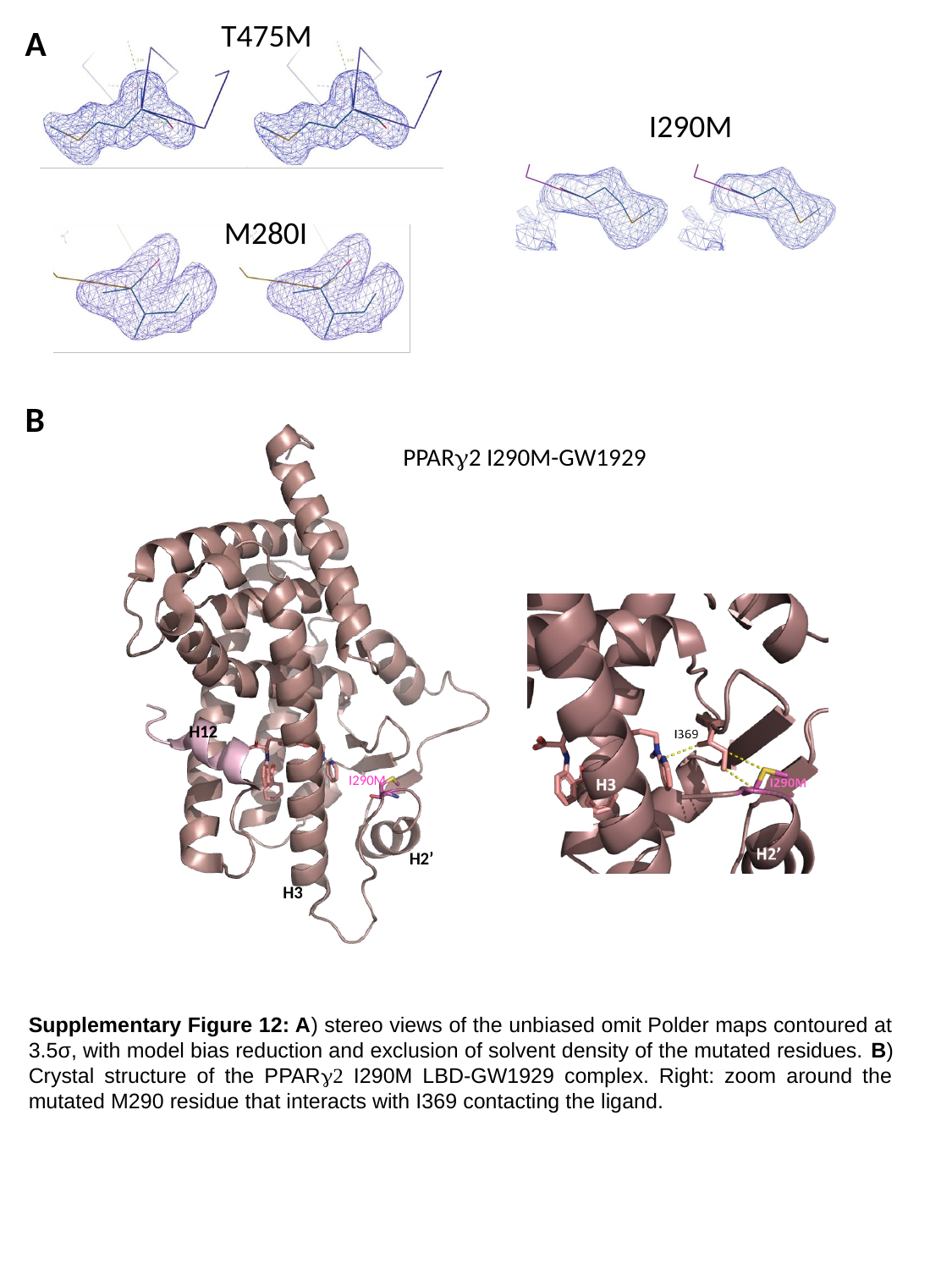

T475M
A
I290M
M280I
B
PPARg2 I290M-GW1929
H12
I290M
H2’
H3
Supplementary Figure 12: A) stereo views of the unbiased omit Polder maps contoured at 3.5σ, with model bias reduction and exclusion of solvent density of the mutated residues. B) Crystal structure of the PPARγ2 I290M LBD-GW1929 complex. Right: zoom around the mutated M290 residue that interacts with I369 contacting the ligand.

## Slide 20
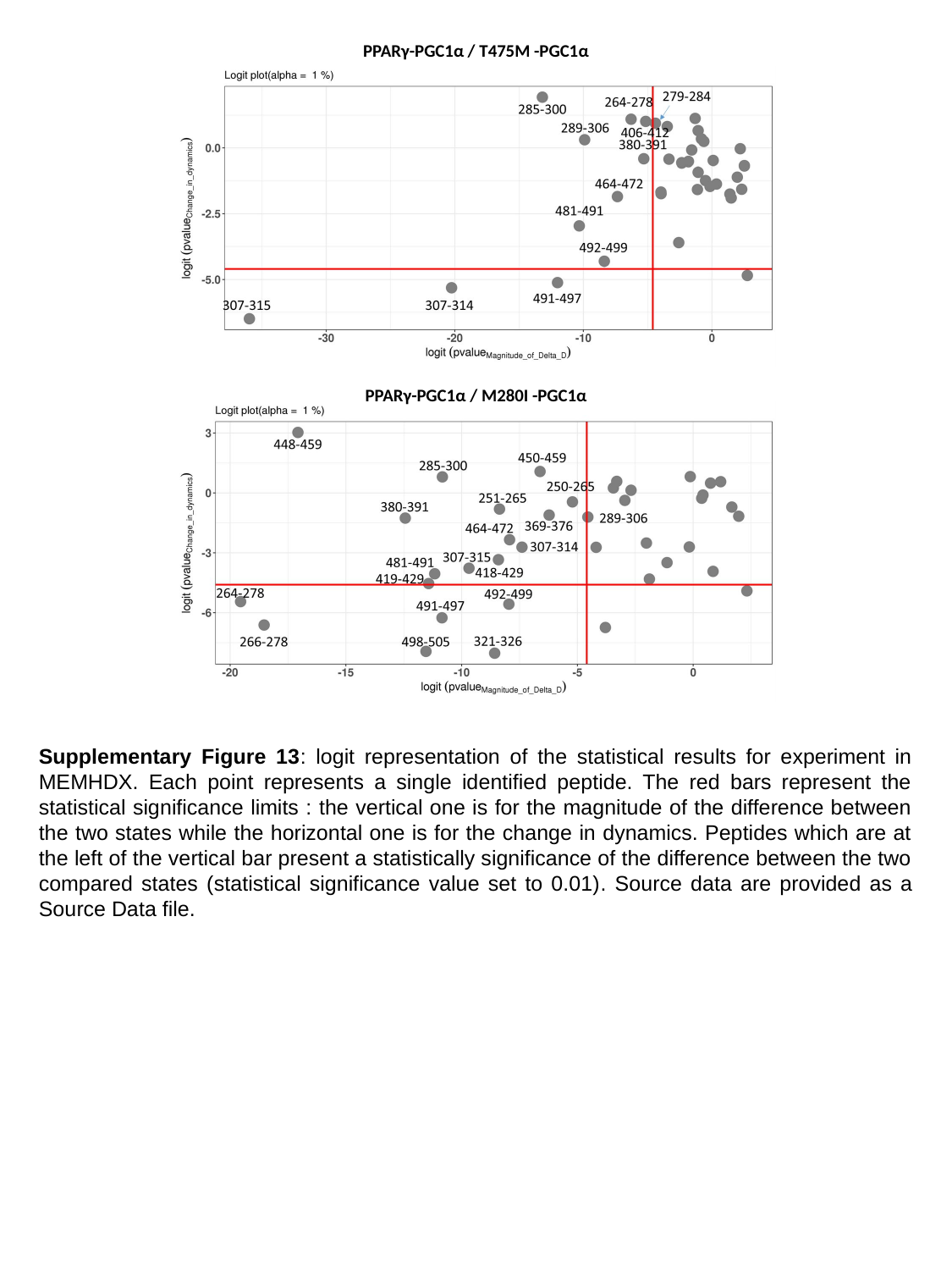

PPARγ-PGC1α / T475M -PGC1α
PPARγ-PGC1α / M280I -PGC1α
Supplementary Figure 13: logit representation of the statistical results for experiment in MEMHDX. Each point represents a single identified peptide. The red bars represent the statistical significance limits : the vertical one is for the magnitude of the difference between the two states while the horizontal one is for the change in dynamics. Peptides which are at the left of the vertical bar present a statistically significance of the difference between the two compared states (statistical significance value set to 0.01). Source data are provided as a Source Data file.

## Slide 21
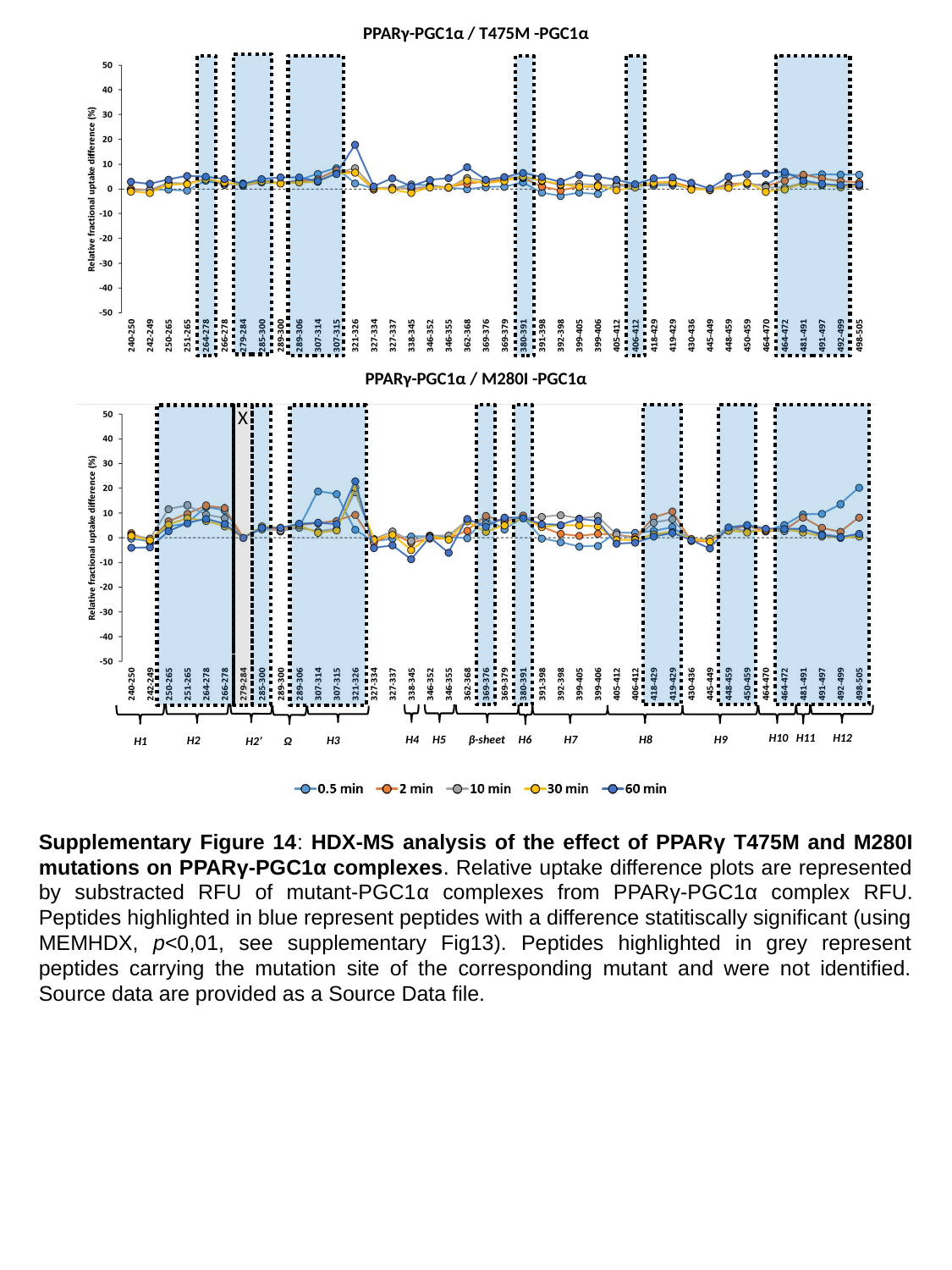

PPARγ-PGC1α / T475M -PGC1α
PPARγ-PGC1α / M280I -PGC1α
X
H12
H10
H11
β-sheet
H6
H7
H8
H9
H4
H5
H2
H3
Ω
H1
H2’
Supplementary Figure 14: HDX-MS analysis of the effect of PPARγ T475M and M280I mutations on PPARγ-PGC1α complexes. Relative uptake difference plots are represented by substracted RFU of mutant-PGC1α complexes from PPARγ-PGC1α complex RFU. Peptides highlighted in blue represent peptides with a difference statitiscally significant (using MEMHDX, p<0,01, see supplementary Fig13). Peptides highlighted in grey represent peptides carrying the mutation site of the corresponding mutant and were not identified. Source data are provided as a Source Data file.

## Slide 22
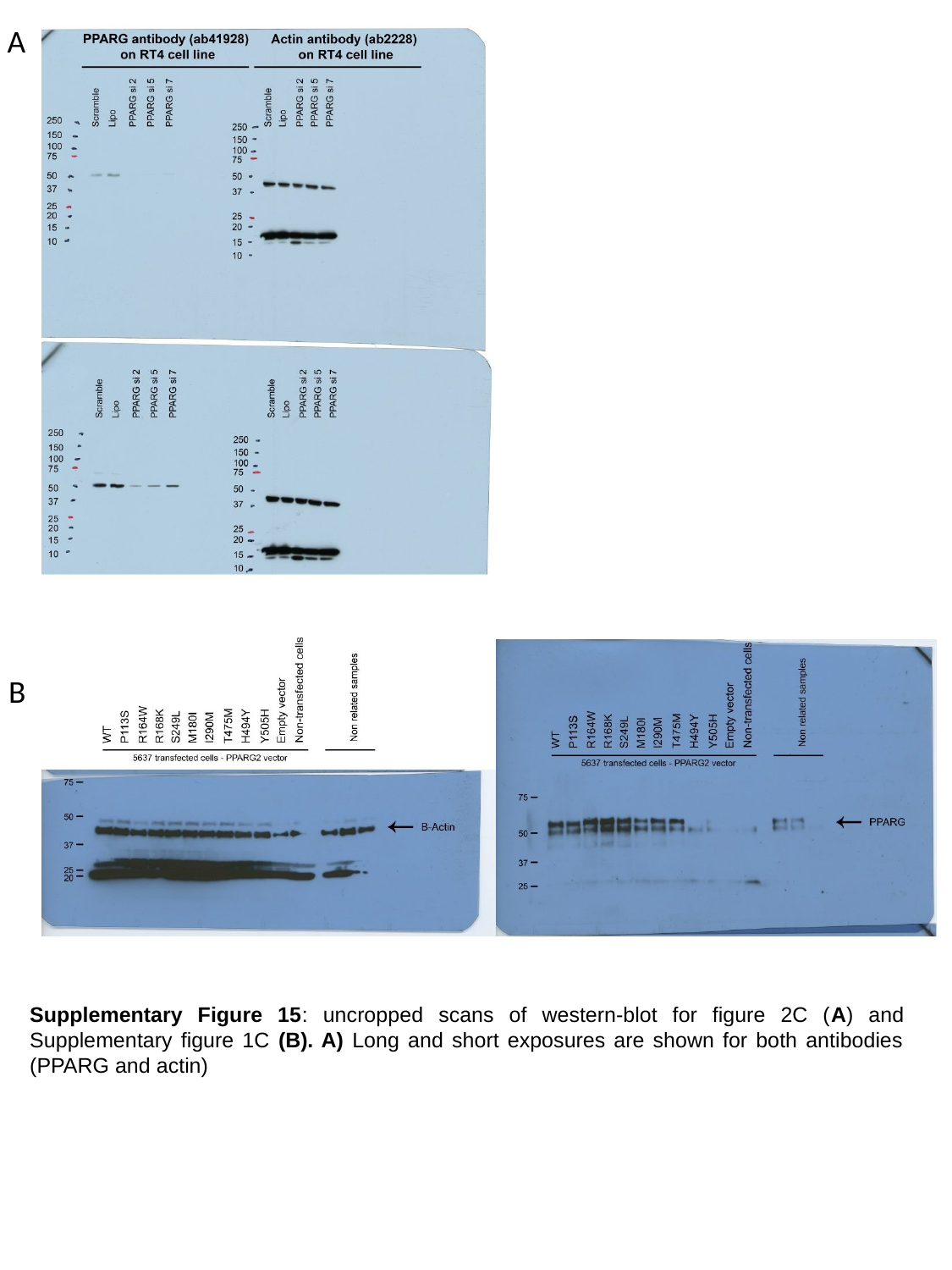

A
B
Supplementary Figure 15: uncropped scans of western-blot for figure 2C (A) and Supplementary figure 1C (B). A) Long and short exposures are shown for both antibodies (PPARG and actin)

## Slide 23
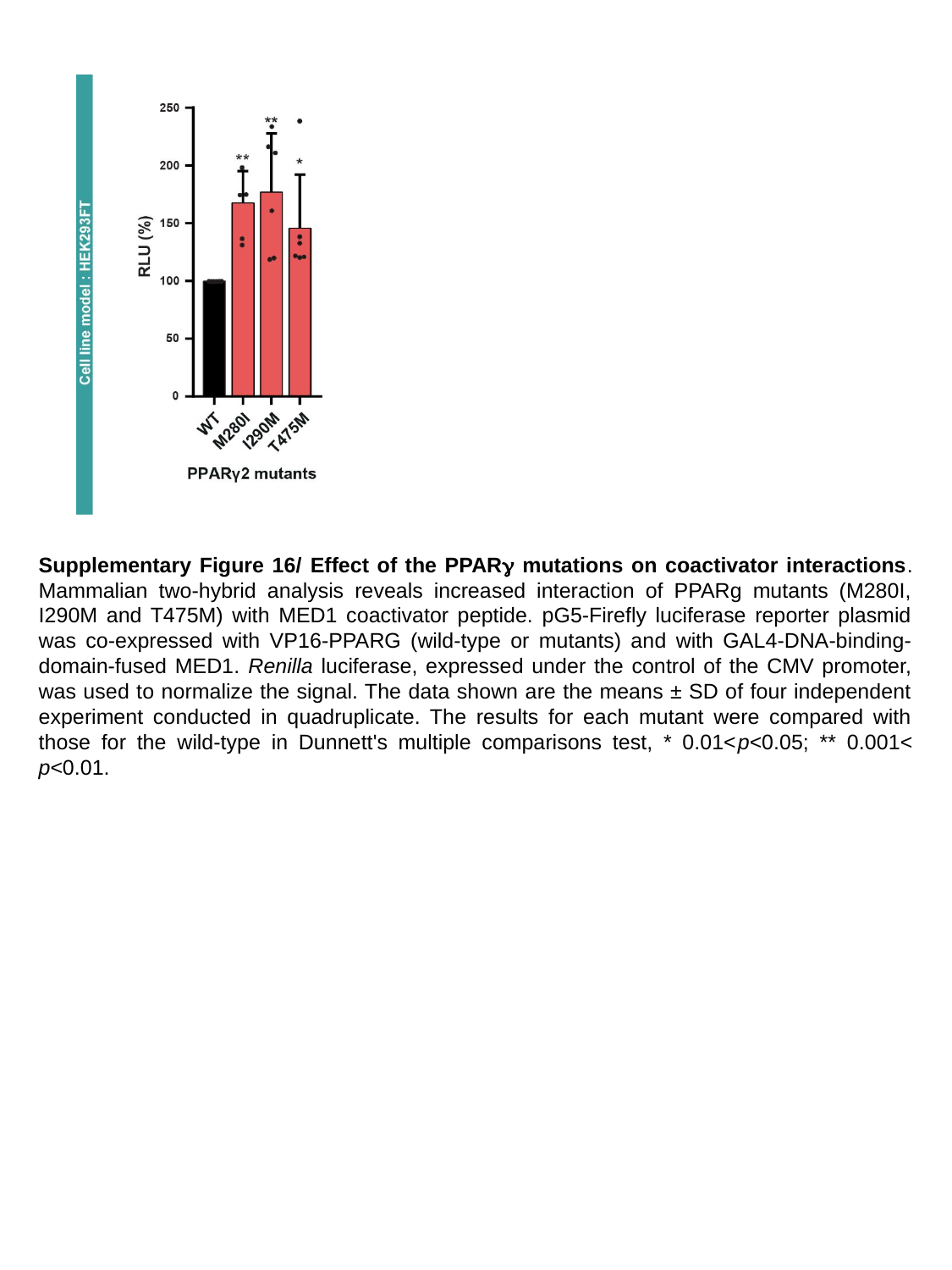

Supplementary Figure 16/ Effect of the PPARγ mutations on coactivator interactions. Mammalian two-hybrid analysis reveals increased interaction of PPARg mutants (M280I, I290M and T475M) with MED1 coactivator peptide. pG5-Firefly luciferase reporter plasmid was co-expressed with VP16-PPARG (wild-type or mutants) and with GAL4-DNA-binding-domain-fused MED1. Renilla luciferase, expressed under the control of the CMV promoter, was used to normalize the signal. The data shown are the means ± SD of four independent experiment conducted in quadruplicate. The results for each mutant were compared with those for the wild-type in Dunnett's multiple comparisons test, * 0.01<p<0.05; ** 0.001< p<0.01.
