# Supplementary material for: Recurrent activating mutations of PPARγ associated with luminal bladder tumors
Source: Nat Commun. 2019 Jan 16;10:253. doi: 10.1038/s41467-018-08157-y (PMC6335423; doi:10.1038/s41467-018-08157-y)
Supplement: Supplementary file 3 — Reporting Summary [file 41467_2018_8157_MOESM3_ESM.pdf]

nature research

Corresponding author(s): Isabelle Bernard-Pierrot
Natacha Rochel

Reporting Summary

Nature Research wishes to improve the reproducibility of the work that we publish. This form provides structure for consistency and transparency in reporting. For further information on Nature Research policies, see Authors & Referees and the Editorial Policy Checklist.

Statistical parameters

When statistical analyses are reported, confirm that the following items are present in the relevant location (e.g. figure legend, table legend, main text, or Methods section).

n/a	Confirmed	
☐	☒	The exact sample size (*n*) for each experimental group/condition, given as a discrete number and unit of measurement
☐	☒	An indication of whether measurements were taken from distinct samples or whether the same sample was measured repeatedly
☐	☒	The statistical test(s) used AND whether they are one- or two-sided<br>*Only common tests should be described solely by name; describe more complex techniques in the Methods section.*
☒	☐	A description of all covariates tested
☐	☒	A description of any assumptions or corrections, such as tests of normality and adjustment for multiple comparisons
☐	☒	A full description of the statistics including central tendency (e.g. means) or other basic estimates (e.g. regression coefficient) AND variation (e.g. standard deviation) or associated estimates of uncertainty (e.g. confidence intervals)
☒	☐	For null hypothesis testing, the test statistic (e.g. *F*, *t*, *r*) with confidence intervals, effect sizes, degrees of freedom and *P* value noted<br>*Give P values as exact values whenever suitable.*
☒	☐	For Bayesian analysis, information on the choice of priors and Markov chain Monte Carlo settings
☒	☐	For hierarchical and complex designs, identification of the appropriate level for tests and full reporting of outcomes
☒	☐	Estimates of effect sizes (e.g. Cohen's *d*, Pearson's *r*), indicating how they were calculated
☐	☒	Clearly defined error bars<br>*State explicitly what error bars represent (e.g. SD, SE, CI)*

Our web collection on statistics for biologists may be useful.

Software and code

Policy information about availability of computer code

Data collection	For all crystal structures, the data were indexed and integrated with XDS and scaled with AIMLESS. The point mutation was built into this experimental crystal structure with CHARMM program version c37b1. All forms were subjected to the PROPKA program to determine the protonation states of titratable residue. Hydrogen atom positions were positioned with the HBUILD46 module of the CHARMM program. For HDX-MS data, deuterium uptake for all identified peptides was checked and validated manually using DynamX 3.0 .
Data analysis	We used Graph Pad Prism software for statistical analysis of the study. Heat maps and lolliplot of PPARG and RXRA mutations were performed using GNU R. The structures were solved by molecular replacement in PHASER and refined with PHENIX and BUSTER with TLS refinement, followed by iterative model building in COOT. All structural figures were prepared with PyMOL . For microscale thermophoresis analysis, NanoTemper Analysis 2.2.4 software was used to fit the data and to determine the Kd. Sedfit software was used to analyse ultracentrifugation data. For SPR analysis, KD and koff were determined using the Biacore T200 Evaluation software. For native MS, data interpretation was performed using MassLynx 4.1. Collision induced unfolding experiments (CIU) were anayzed using the open-source CIU-Suite software. HDX-MS results were statistically validated using Mixed-Effects Model for HDX experiments (MEMHDX).

For manuscripts utilizing custom algorithms or software that are central to the research but not yet described in published literature, software must be made available to editors/reviewers upon request. We strongly encourage code deposition in a community repository (e.g. GitHub). See the Nature Research guidelines for submitting code & software for further information.

Data

Policy information about availability of data

All manuscripts must include a data availability statement. This statement should provide the following information, where applicable:

- Accession codes, unique identifiers, or web links for publicly available datasets
- A list of figures that have associated raw data
- A description of any restrictions on data availability

Atomic coordinates and related structure factors have been deposited in the Protein Data Bank with accession codes: 6FZY (PPARγ T475M apo), 6FZF (PPARγ T475M-GW1929-PGC1α), 6FZJ (PPARγ M280I-GW1929-PGC1α), 6FZG (PPARγ I290M-GW1929) and 6FZP (PPARγ-GW1929-PGC1α). The source data underlying Figs 1-3 and Supplementary Figs 1, 13 and 14 are provided as a Source Data file. All other data supporting the findings of this study are available from the corresponding authors on reasonable request.

Field-specific reporting

Please select the best fit for your research. If you are not sure, read the appropriate sections before making your selection.

☒ Life sciences ☐ Behavioural & social sciences ☐ Ecological, evolutionary & environmental sciences

For a reference copy of the document with all sections, see nature.com/authors/policies/ReportingSummary-flat.pdf

Life sciences study design

All studies must disclose on these points even when the disclosure is negative.

Sample size	We did not perform any sample size calculation. We have studied RXRA and PPARG mutations in 4 independent series encompassing in total 814 bladder tumors that should allow a quite accurate determination of the frequency of the mutations .
Data exclusions	no data exclusion
Replication	The sequencing of RXRA and PPARG was performed using two different methods (sanger sequencing VS NGS) in independent series.<br>The fonctionnal and biochemical experiments were repeated at least three times
Randomization	Not relevant to our study based only on in vitro data
Blinding	For some experiments (transfection of 5637 cells with PPARG mutants to study the effects on PPARG target genes expression, transfection of RT4 cells with PPARG siRNA to evaluate the effects on cell growth), transfection and collection of the cells/analysis of data were performed by two different persons so that the analysis were done in a blind way. For the other biochemical analysis, no blinding was performed to study the different mutants.

Reporting for specific materials, systems and methods

Materials & experimental systems

n/a	Involved in the study
☒	☐ Unique biological materials
☐	☒ Antibodies
☐	☒ Eukaryotic cell lines
☒	☐ Palaeontology
☒	☐ Animals and other organisms
☒	☐ Human research participants

Methods

n/a	Involved in the study
☒	☐ ChIP-seq
☒	☐ Flow cytometry
☒	☐ MRI-based neuroimaging

Antibodies

Antibodies used	Anti-PPAR gamma 1+2 antibody [A3409A]: Abcam #ab41928, lot GR26030-35<br>Monoclonal Anti-β-Actin antibody: Sigma Aldrich #A2228, clone AC-74, lot 066m486Ov
Validation	Anti-PPAR gamma 1+2 antibody [A3409A]: Suitable for WB<br>We verified that after a PPARG knock-down using siRNA the observed band in WB did decrease accordingly with mRNA level decreased suggesting a good specificity of the antibody.

Monoclonal Anti-β-Actin antibody: Monoclonal mouse anti-actin antibody was used as a loading control for western blot. We did not address the specificity of the antibody.

Eukaryotic cell lines

Policy information about cell lines

Cell line source(s)	The HEK293FT human cell line and the RT4 and 5637 human bladder tumor-derived cell lines were obtained from DSMZ (Heidelberg, Germany)
Authentication	The identity of RT4 and 5637 cell lines used was checked by analyzing genomic alterations with comparative genomic hybridization arrays (CGH array), by analyzing their transcriptomic profile (Affymetrix U133plus2 array) and by analyzing FGFR3 fusions and mutations. The results obtained were compared with the initial description of the cells. HEK293FT cells were used for reporter assays and mamalian two-hybrid assays. These two kind of test being quite independant of the cell line identity , we did not check further the identity of these cells.
Mycoplasma contamination	We routinely check for mycoplasma contamination and all the cell lines used were negative for mycoplasma contamination.
Commonly misidentified lines (See ICLAC register)	not relevant here
