# Supplementary material for: Recurrent activating mutations of PPARγ associated with luminal bladder tumors
Source: Nat Commun. 2019 Jan 16;10:253. doi: 10.1038/s41467-018-08157-y (PMC6335423; doi:10.1038/s41467-018-08157-y)
Supplement: Supplementary file 4 — Source Data [file 41467_2018_8157_MOESM4_ESM.zip › source data-14122018/Realtive_uptakesU_wt-PGC1_T475M-PGC1- SuppFig13/index.htm]

DynamX Export
