# Supplementary figures and images for: Recurrent activating mutations of PPARγ associated with luminal bladder tumors
Source: Nat Commun. 2019 Jan 16;10:253. doi: 10.1038/s41467-018-08157-y (PMC6335423; doi:10.1038/s41467-018-08157-y)

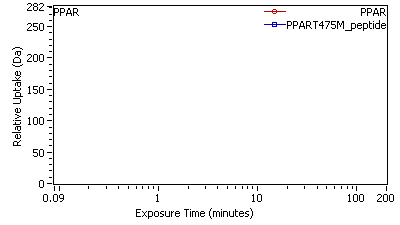

Supplement: Supplementary file 4 — Source Data [file 41467_2018_8157_MOESM4_ESM.zip › source data-14122018/Realtive_uptakesU_wt-PGC1_T475M-PGC1- SuppFig13/01 PPAR[2-296] GSSHHHHHHSSGLVPR.png]

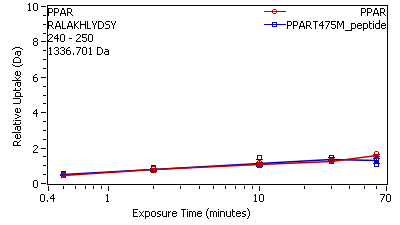

Supplement: Supplementary file 4 — Source Data [file 41467_2018_8157_MOESM4_ESM.zip › source data-14122018/Realtive_uptakesU_wt-PGC1_T475M-PGC1- SuppFig13/02 PPAR[240-250] RALAKHLYDSY.png]

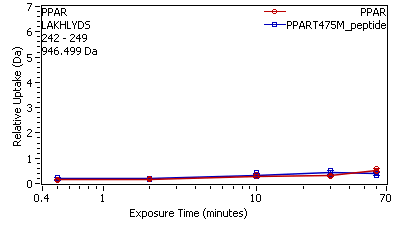

Supplement: Supplementary file 4 — Source Data [file 41467_2018_8157_MOESM4_ESM.zip › source data-14122018/Realtive_uptakesU_wt-PGC1_T475M-PGC1- SuppFig13/03 PPAR[242-249] LAKHLYDS.png]

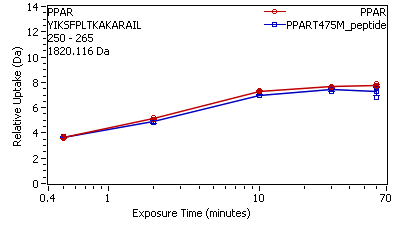

Supplement: Supplementary file 4 — Source Data [file 41467_2018_8157_MOESM4_ESM.zip › source data-14122018/Realtive_uptakesU_wt-PGC1_T475M-PGC1- SuppFig13/04 PPAR[250-265] YIKSFPLTKAKARAIL.png]

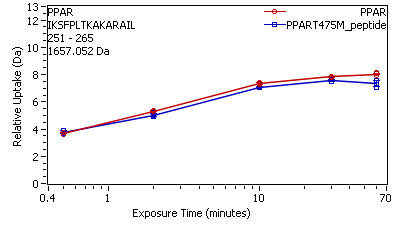

Supplement: Supplementary file 4 — Source Data [file 41467_2018_8157_MOESM4_ESM.zip › source data-14122018/Realtive_uptakesU_wt-PGC1_T475M-PGC1- SuppFig13/05 PPAR[251-265] IKSFPLTKAKARAIL.png]

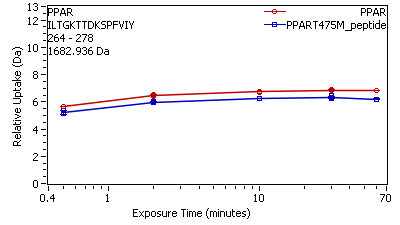

Supplement: Supplementary file 4 — Source Data [file 41467_2018_8157_MOESM4_ESM.zip › source data-14122018/Realtive_uptakesU_wt-PGC1_T475M-PGC1- SuppFig13/06 PPAR[264-278] ILTGKTTDKSPFVIY.png]

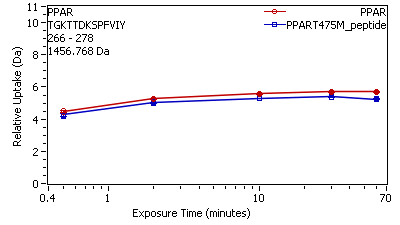

Supplement: Supplementary file 4 — Source Data [file 41467_2018_8157_MOESM4_ESM.zip › source data-14122018/Realtive_uptakesU_wt-PGC1_T475M-PGC1- SuppFig13/07 PPAR[266-278] TGKTTDKSPFVIY.png]

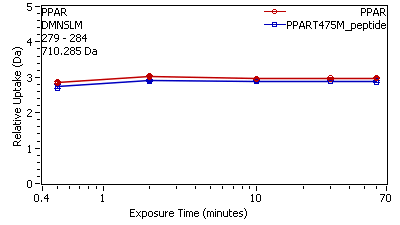

Supplement: Supplementary file 4 — Source Data [file 41467_2018_8157_MOESM4_ESM.zip › source data-14122018/Realtive_uptakesU_wt-PGC1_T475M-PGC1- SuppFig13/08 PPAR[279-284] DMNSLM.png]

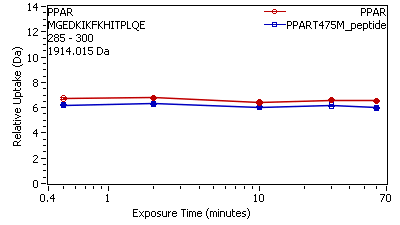

Supplement: Supplementary file 4 — Source Data [file 41467_2018_8157_MOESM4_ESM.zip › source data-14122018/Realtive_uptakesU_wt-PGC1_T475M-PGC1- SuppFig13/09 PPAR[285-300] MGEDKIKFKHITPLQE.png]

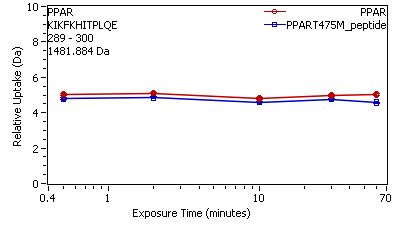

Supplement: Supplementary file 4 — Source Data [file 41467_2018_8157_MOESM4_ESM.zip › source data-14122018/Realtive_uptakesU_wt-PGC1_T475M-PGC1- SuppFig13/10 PPAR[289-300] KIKFKHITPLQE.png]

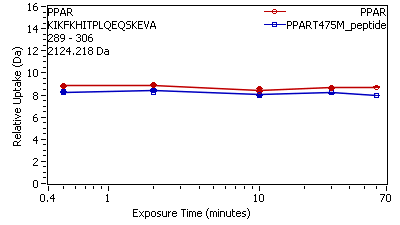

Supplement: Supplementary file 4 — Source Data [file 41467_2018_8157_MOESM4_ESM.zip › source data-14122018/Realtive_uptakesU_wt-PGC1_T475M-PGC1- SuppFig13/11 PPAR[289-306] KIKFKHITPLQEQSKE.png]

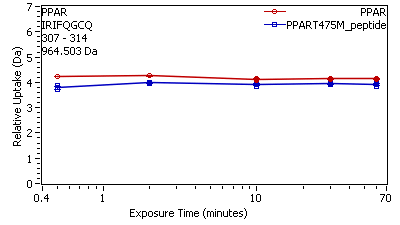

Supplement: Supplementary file 4 — Source Data [file 41467_2018_8157_MOESM4_ESM.zip › source data-14122018/Realtive_uptakesU_wt-PGC1_T475M-PGC1- SuppFig13/12 PPAR[307-314] IRIFQGCQ.png]

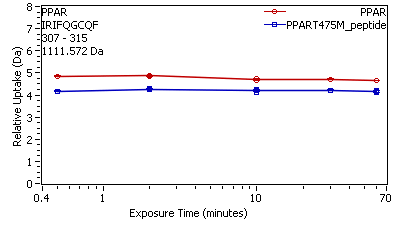

Supplement: Supplementary file 4 — Source Data [file 41467_2018_8157_MOESM4_ESM.zip › source data-14122018/Realtive_uptakesU_wt-PGC1_T475M-PGC1- SuppFig13/13 PPAR[307-315] IRIFQGCQF.png]

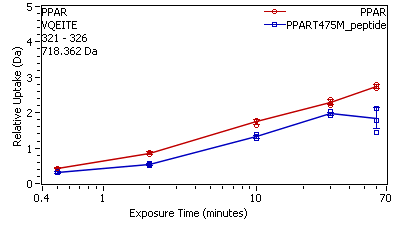

Supplement: Supplementary file 4 — Source Data [file 41467_2018_8157_MOESM4_ESM.zip › source data-14122018/Realtive_uptakesU_wt-PGC1_T475M-PGC1- SuppFig13/14 PPAR[321-326] VQEITE.png]

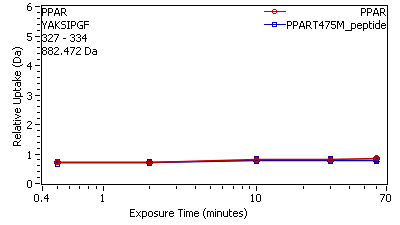

Supplement: Supplementary file 4 — Source Data [file 41467_2018_8157_MOESM4_ESM.zip › source data-14122018/Realtive_uptakesU_wt-PGC1_T475M-PGC1- SuppFig13/15 PPAR[327-334] YAKSIPGF.png]

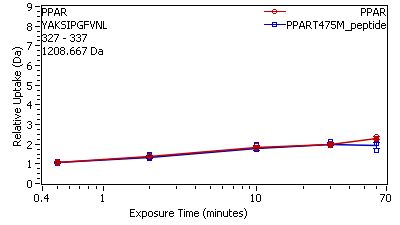

Supplement: Supplementary file 4 — Source Data [file 41467_2018_8157_MOESM4_ESM.zip › source data-14122018/Realtive_uptakesU_wt-PGC1_T475M-PGC1- SuppFig13/16 PPAR[327-337] YAKSIPGFVNL.png]

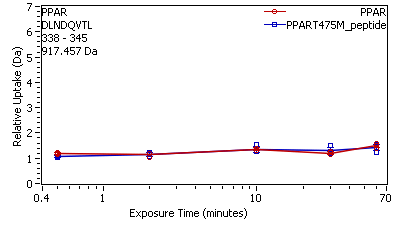

Supplement: Supplementary file 4 — Source Data [file 41467_2018_8157_MOESM4_ESM.zip › source data-14122018/Realtive_uptakesU_wt-PGC1_T475M-PGC1- SuppFig13/17 PPAR[338-345] DLNDQVTL.png]

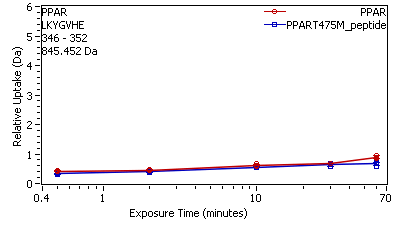

Supplement: Supplementary file 4 — Source Data [file 41467_2018_8157_MOESM4_ESM.zip › source data-14122018/Realtive_uptakesU_wt-PGC1_T475M-PGC1- SuppFig13/18 PPAR[346-352] LKYGVHE.png]

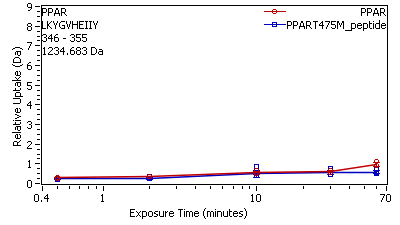

Supplement: Supplementary file 4 — Source Data [file 41467_2018_8157_MOESM4_ESM.zip › source data-14122018/Realtive_uptakesU_wt-PGC1_T475M-PGC1- SuppFig13/19 PPAR[346-355] LKYGVHEIIY.png]

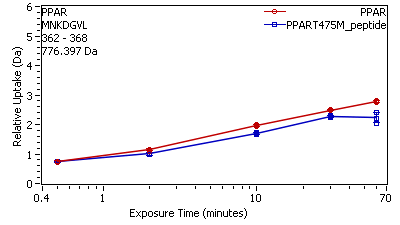

Supplement: Supplementary file 4 — Source Data [file 41467_2018_8157_MOESM4_ESM.zip › source data-14122018/Realtive_uptakesU_wt-PGC1_T475M-PGC1- SuppFig13/20 PPAR[362-368] MNKDGVL.png]

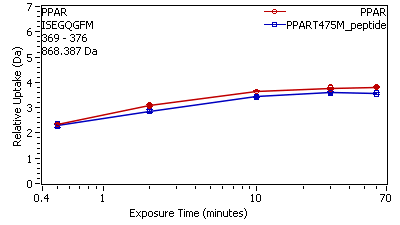

Supplement: Supplementary file 4 — Source Data [file 41467_2018_8157_MOESM4_ESM.zip › source data-14122018/Realtive_uptakesU_wt-PGC1_T475M-PGC1- SuppFig13/21 PPAR[369-376] ISEGQGFM.png]

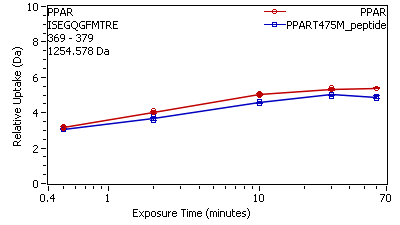

Supplement: Supplementary file 4 — Source Data [file 41467_2018_8157_MOESM4_ESM.zip › source data-14122018/Realtive_uptakesU_wt-PGC1_T475M-PGC1- SuppFig13/22 PPAR[369-379] ISEGQGFMTRE.png]

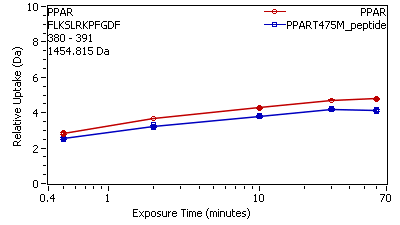

Supplement: Supplementary file 4 — Source Data [file 41467_2018_8157_MOESM4_ESM.zip › source data-14122018/Realtive_uptakesU_wt-PGC1_T475M-PGC1- SuppFig13/23 PPAR[380-391] FLKSLRKPFGDF.png]

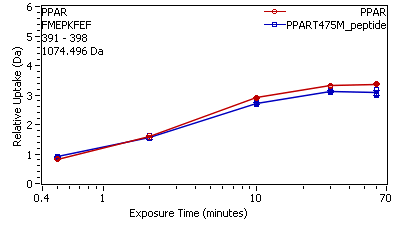

Supplement: Supplementary file 4 — Source Data [file 41467_2018_8157_MOESM4_ESM.zip › source data-14122018/Realtive_uptakesU_wt-PGC1_T475M-PGC1- SuppFig13/24 PPAR[391-398] FMEPKFEF.png]

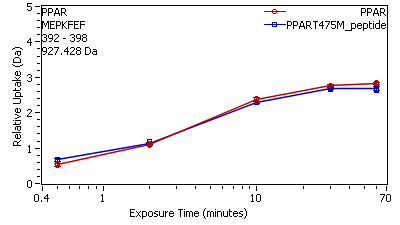

Supplement: Supplementary file 4 — Source Data [file 41467_2018_8157_MOESM4_ESM.zip › source data-14122018/Realtive_uptakesU_wt-PGC1_T475M-PGC1- SuppFig13/25 PPAR[392-398] MEPKFEF.png]

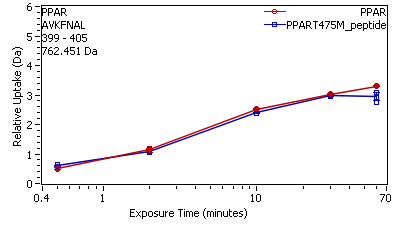

Supplement: Supplementary file 4 — Source Data [file 41467_2018_8157_MOESM4_ESM.zip › source data-14122018/Realtive_uptakesU_wt-PGC1_T475M-PGC1- SuppFig13/26 PPAR[399-405] AVKFNAL.png]

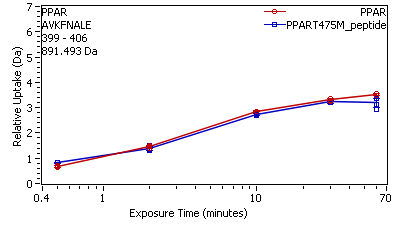

Supplement: Supplementary file 4 — Source Data [file 41467_2018_8157_MOESM4_ESM.zip › source data-14122018/Realtive_uptakesU_wt-PGC1_T475M-PGC1- SuppFig13/27 PPAR[399-406] AVKFNALE.png]

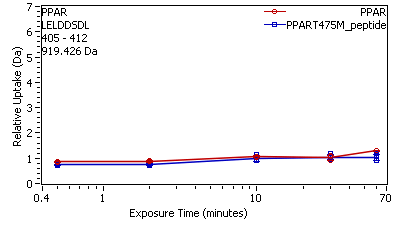

Supplement: Supplementary file 4 — Source Data [file 41467_2018_8157_MOESM4_ESM.zip › source data-14122018/Realtive_uptakesU_wt-PGC1_T475M-PGC1- SuppFig13/28 PPAR[405-412] LELDDSDL.png]

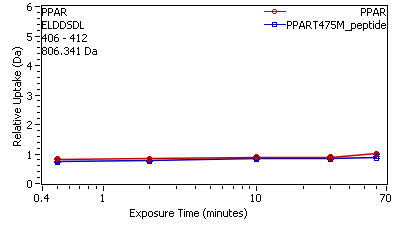

Supplement: Supplementary file 4 — Source Data [file 41467_2018_8157_MOESM4_ESM.zip › source data-14122018/Realtive_uptakesU_wt-PGC1_T475M-PGC1- SuppFig13/29 PPAR[406-412] ELDDSDL.png]

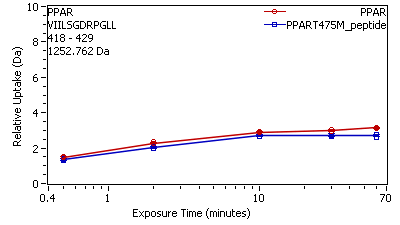

Supplement: Supplementary file 4 — Source Data [file 41467_2018_8157_MOESM4_ESM.zip › source data-14122018/Realtive_uptakesU_wt-PGC1_T475M-PGC1- SuppFig13/30 PPAR[418-429] VIILSGDRPGLL.png]

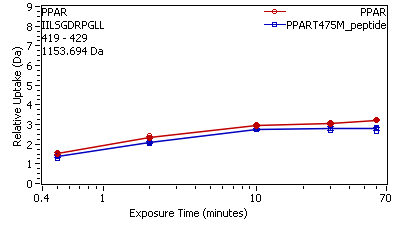

Supplement: Supplementary file 4 — Source Data [file 41467_2018_8157_MOESM4_ESM.zip › source data-14122018/Realtive_uptakesU_wt-PGC1_T475M-PGC1- SuppFig13/31 PPAR[419-429] IILSGDRPGLL.png]

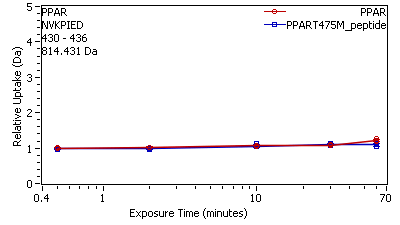

Supplement: Supplementary file 4 — Source Data [file 41467_2018_8157_MOESM4_ESM.zip › source data-14122018/Realtive_uptakesU_wt-PGC1_T475M-PGC1- SuppFig13/32 PPAR[430-436] NVKPIED.png]

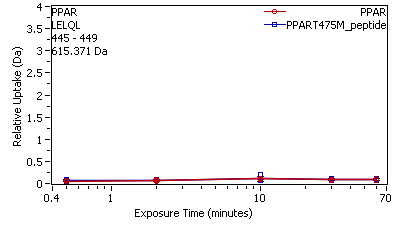

Supplement: Supplementary file 4 — Source Data [file 41467_2018_8157_MOESM4_ESM.zip › source data-14122018/Realtive_uptakesU_wt-PGC1_T475M-PGC1- SuppFig13/33 PPAR[445-449] LELQL.png]

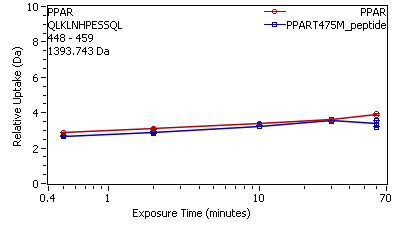

Supplement: Supplementary file 4 — Source Data [file 41467_2018_8157_MOESM4_ESM.zip › source data-14122018/Realtive_uptakesU_wt-PGC1_T475M-PGC1- SuppFig13/34 PPAR[448-459] QLKLNHPESSQL.png]

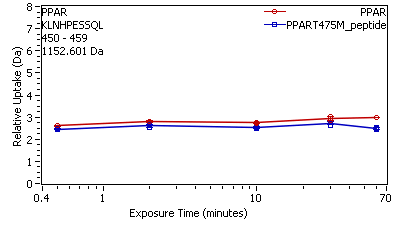

Supplement: Supplementary file 4 — Source Data [file 41467_2018_8157_MOESM4_ESM.zip › source data-14122018/Realtive_uptakesU_wt-PGC1_T475M-PGC1- SuppFig13/35 PPAR[450-459] KLNHPESSQL.png]

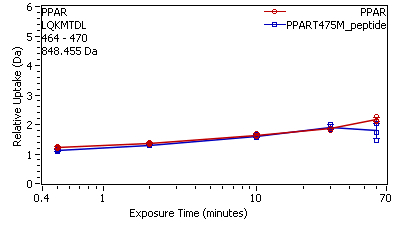

Supplement: Supplementary file 4 — Source Data [file 41467_2018_8157_MOESM4_ESM.zip › source data-14122018/Realtive_uptakesU_wt-PGC1_T475M-PGC1- SuppFig13/36 PPAR[464-470] LQKMTDL.png]

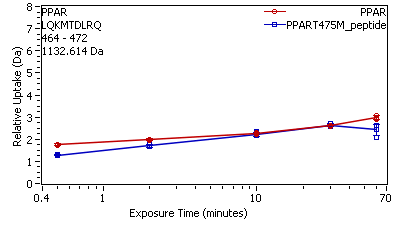

Supplement: Supplementary file 4 — Source Data [file 41467_2018_8157_MOESM4_ESM.zip › source data-14122018/Realtive_uptakesU_wt-PGC1_T475M-PGC1- SuppFig13/37 PPAR[464-472] LQKMTDLRQ.png]

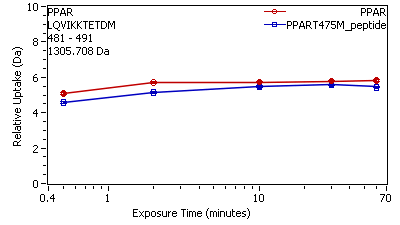

Supplement: Supplementary file 4 — Source Data [file 41467_2018_8157_MOESM4_ESM.zip › source data-14122018/Realtive_uptakesU_wt-PGC1_T475M-PGC1- SuppFig13/38 PPAR[481-491] LQVIKKTETDM.png]

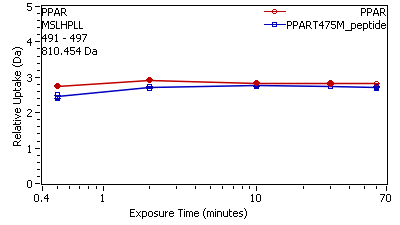

Supplement: Supplementary file 4 — Source Data [file 41467_2018_8157_MOESM4_ESM.zip › source data-14122018/Realtive_uptakesU_wt-PGC1_T475M-PGC1- SuppFig13/39 PPAR[491-497] MSLHPLL.png]

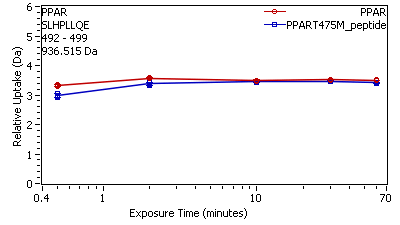

Supplement: Supplementary file 4 — Source Data [file 41467_2018_8157_MOESM4_ESM.zip › source data-14122018/Realtive_uptakesU_wt-PGC1_T475M-PGC1- SuppFig13/40 PPAR[492-499] SLHPLLQE.png]

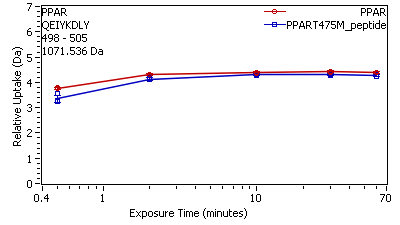

Supplement: Supplementary file 4 — Source Data [file 41467_2018_8157_MOESM4_ESM.zip › source data-14122018/Realtive_uptakesU_wt-PGC1_T475M-PGC1- SuppFig13/41 PPAR[498-505] QEIYKDLY.png]

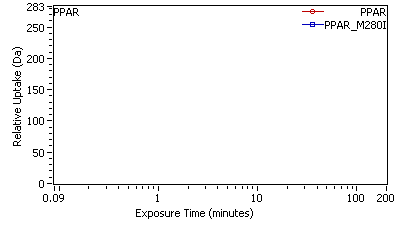

Supplement: Supplementary file 4 — Source Data [file 41467_2018_8157_MOESM4_ESM.zip › source data-14122018/Realtive_uptakes_wt-PGC1_M280I-PGC1-SupFig14/01 PPAR[2-297] MGSSHHHHHHSSGLVP.png]

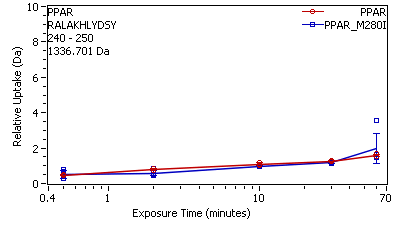

Supplement: Supplementary file 4 — Source Data [file 41467_2018_8157_MOESM4_ESM.zip › source data-14122018/Realtive_uptakes_wt-PGC1_M280I-PGC1-SupFig14/02 PPAR[240-250] RALAKHLYDSY.png]

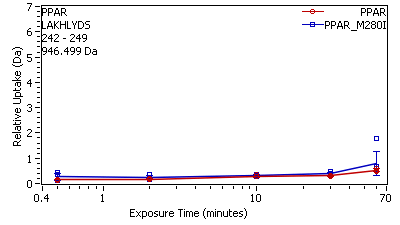

Supplement: Supplementary file 4 — Source Data [file 41467_2018_8157_MOESM4_ESM.zip › source data-14122018/Realtive_uptakes_wt-PGC1_M280I-PGC1-SupFig14/03 PPAR[242-249] LAKHLYDS.png]

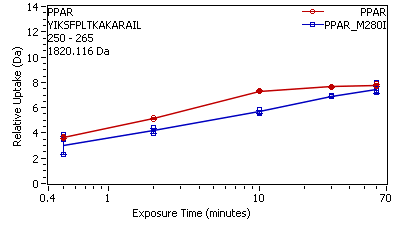

Supplement: Supplementary file 4 — Source Data [file 41467_2018_8157_MOESM4_ESM.zip › source data-14122018/Realtive_uptakes_wt-PGC1_M280I-PGC1-SupFig14/04 PPAR[250-265] YIKSFPLTKAKARAIL.png]

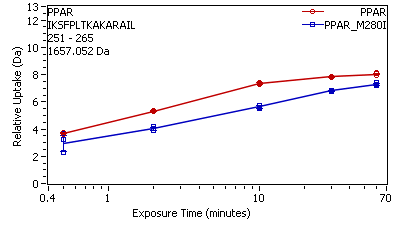

Supplement: Supplementary file 4 — Source Data [file 41467_2018_8157_MOESM4_ESM.zip › source data-14122018/Realtive_uptakes_wt-PGC1_M280I-PGC1-SupFig14/05 PPAR[251-265] IKSFPLTKAKARAIL.png]

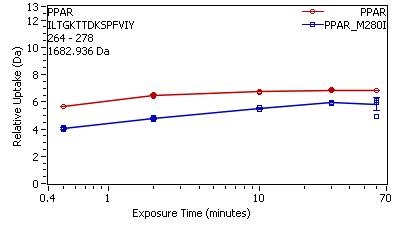

Supplement: Supplementary file 4 — Source Data [file 41467_2018_8157_MOESM4_ESM.zip › source data-14122018/Realtive_uptakes_wt-PGC1_M280I-PGC1-SupFig14/06 PPAR[264-278] ILTGKTTDKSPFVIY.png]

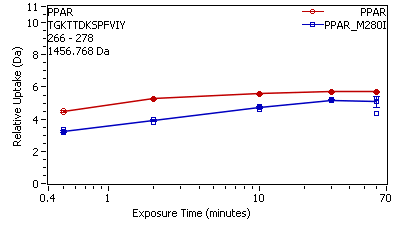

Supplement: Supplementary file 4 — Source Data [file 41467_2018_8157_MOESM4_ESM.zip › source data-14122018/Realtive_uptakes_wt-PGC1_M280I-PGC1-SupFig14/07 PPAR[266-278] TGKTTDKSPFVIY.png]

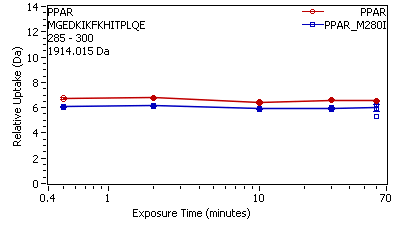

Supplement: Supplementary file 4 — Source Data [file 41467_2018_8157_MOESM4_ESM.zip › source data-14122018/Realtive_uptakes_wt-PGC1_M280I-PGC1-SupFig14/08 PPAR[285-300] MGEDKIKFKHITPLQE.png]

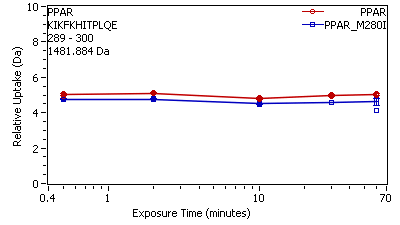

Supplement: Supplementary file 4 — Source Data [file 41467_2018_8157_MOESM4_ESM.zip › source data-14122018/Realtive_uptakes_wt-PGC1_M280I-PGC1-SupFig14/09 PPAR[289-300] KIKFKHITPLQE.png]

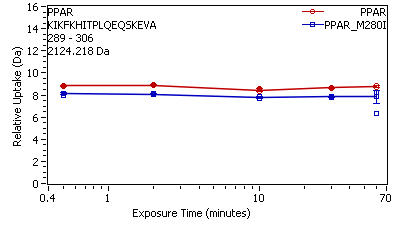

Supplement: Supplementary file 4 — Source Data [file 41467_2018_8157_MOESM4_ESM.zip › source data-14122018/Realtive_uptakes_wt-PGC1_M280I-PGC1-SupFig14/10 PPAR[289-306] KIKFKHITPLQEQSKE.png]

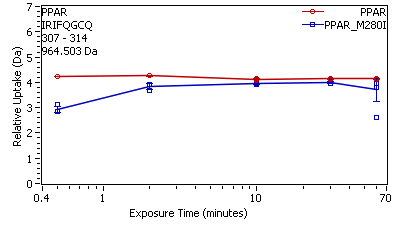

Supplement: Supplementary file 4 — Source Data [file 41467_2018_8157_MOESM4_ESM.zip › source data-14122018/Realtive_uptakes_wt-PGC1_M280I-PGC1-SupFig14/11 PPAR[307-314] IRIFQGCQ.png]

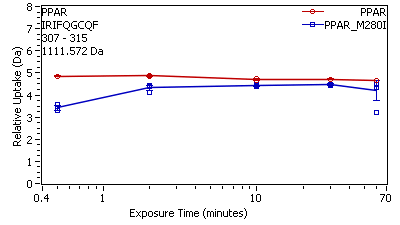

Supplement: Supplementary file 4 — Source Data [file 41467_2018_8157_MOESM4_ESM.zip › source data-14122018/Realtive_uptakes_wt-PGC1_M280I-PGC1-SupFig14/12 PPAR[307-315] IRIFQGCQF.png]

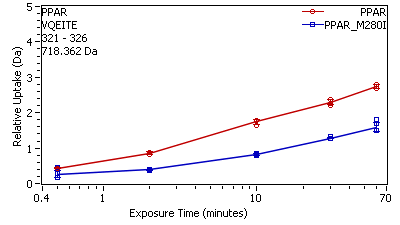

Supplement: Supplementary file 4 — Source Data [file 41467_2018_8157_MOESM4_ESM.zip › source data-14122018/Realtive_uptakes_wt-PGC1_M280I-PGC1-SupFig14/13 PPAR[321-326] VQEITE.png]

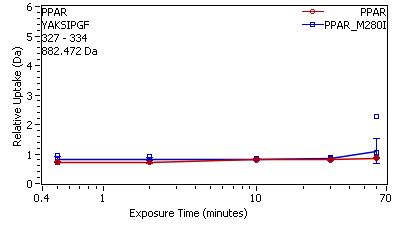

Supplement: Supplementary file 4 — Source Data [file 41467_2018_8157_MOESM4_ESM.zip › source data-14122018/Realtive_uptakes_wt-PGC1_M280I-PGC1-SupFig14/14 PPAR[327-334] YAKSIPGF.png]

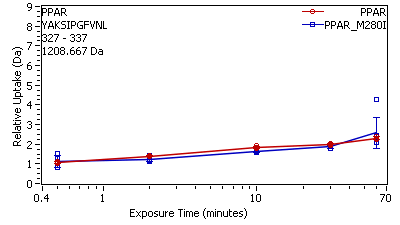

Supplement: Supplementary file 4 — Source Data [file 41467_2018_8157_MOESM4_ESM.zip › source data-14122018/Realtive_uptakes_wt-PGC1_M280I-PGC1-SupFig14/15 PPAR[327-337] YAKSIPGFVNL.png]

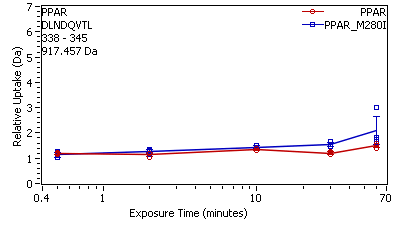

Supplement: Supplementary file 4 — Source Data [file 41467_2018_8157_MOESM4_ESM.zip › source data-14122018/Realtive_uptakes_wt-PGC1_M280I-PGC1-SupFig14/16 PPAR[338-345] DLNDQVTL.png]

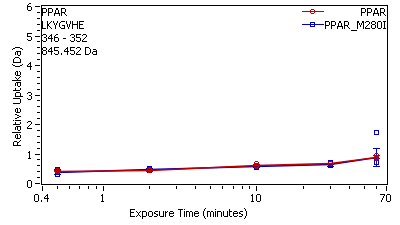

Supplement: Supplementary file 4 — Source Data [file 41467_2018_8157_MOESM4_ESM.zip › source data-14122018/Realtive_uptakes_wt-PGC1_M280I-PGC1-SupFig14/17 PPAR[346-352] LKYGVHE.png]

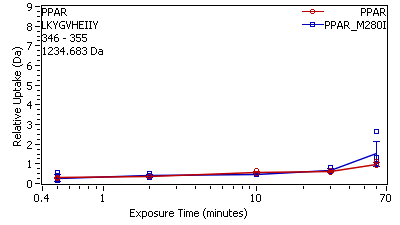

Supplement: Supplementary file 4 — Source Data [file 41467_2018_8157_MOESM4_ESM.zip › source data-14122018/Realtive_uptakes_wt-PGC1_M280I-PGC1-SupFig14/18 PPAR[346-355] LKYGVHEIIY.png]

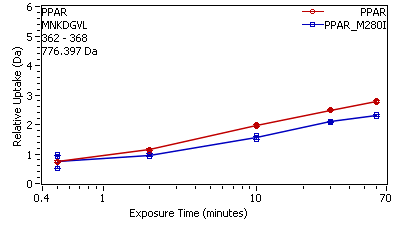

Supplement: Supplementary file 4 — Source Data [file 41467_2018_8157_MOESM4_ESM.zip › source data-14122018/Realtive_uptakes_wt-PGC1_M280I-PGC1-SupFig14/19 PPAR[362-368] MNKDGVL.png]

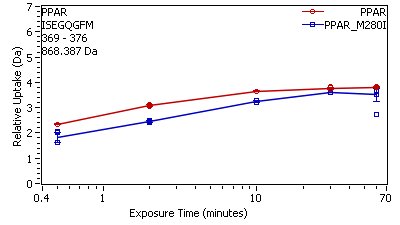

Supplement: Supplementary file 4 — Source Data [file 41467_2018_8157_MOESM4_ESM.zip › source data-14122018/Realtive_uptakes_wt-PGC1_M280I-PGC1-SupFig14/20 PPAR[369-376] ISEGQGFM.png]

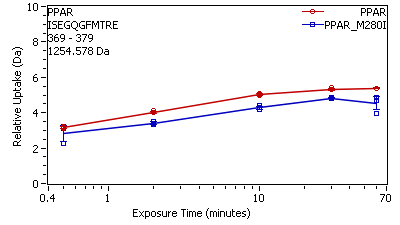

Supplement: Supplementary file 4 — Source Data [file 41467_2018_8157_MOESM4_ESM.zip › source data-14122018/Realtive_uptakes_wt-PGC1_M280I-PGC1-SupFig14/21 PPAR[369-379] ISEGQGFMTRE.png]

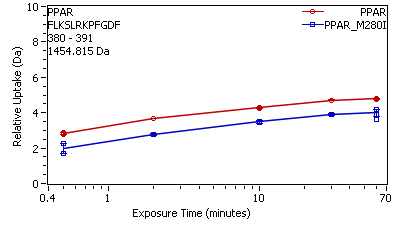

Supplement: Supplementary file 4 — Source Data [file 41467_2018_8157_MOESM4_ESM.zip › source data-14122018/Realtive_uptakes_wt-PGC1_M280I-PGC1-SupFig14/22 PPAR[380-391] FLKSLRKPFGDF.png]

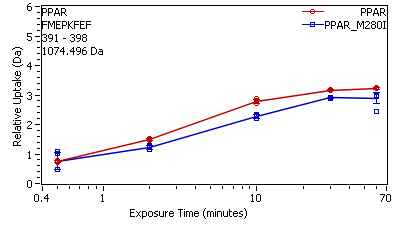

Supplement: Supplementary file 4 — Source Data [file 41467_2018_8157_MOESM4_ESM.zip › source data-14122018/Realtive_uptakes_wt-PGC1_M280I-PGC1-SupFig14/23 PPAR[391-398] FMEPKFEF.png]

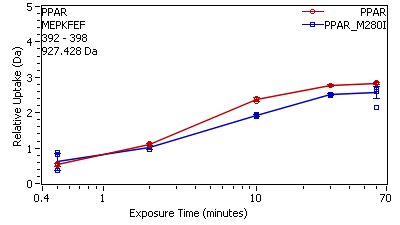

Supplement: Supplementary file 4 — Source Data [file 41467_2018_8157_MOESM4_ESM.zip › source data-14122018/Realtive_uptakes_wt-PGC1_M280I-PGC1-SupFig14/24 PPAR[392-398] MEPKFEF.png]

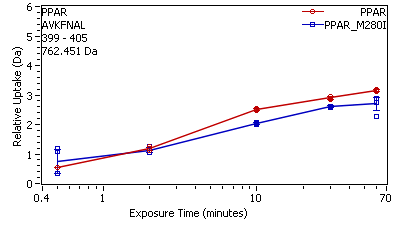

Supplement: Supplementary file 4 — Source Data [file 41467_2018_8157_MOESM4_ESM.zip › source data-14122018/Realtive_uptakes_wt-PGC1_M280I-PGC1-SupFig14/25 PPAR[399-405] AVKFNAL.png]

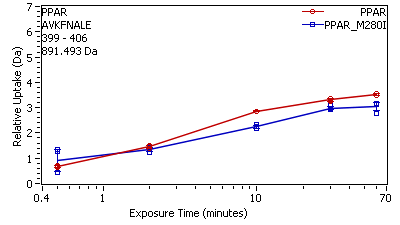

Supplement: Supplementary file 4 — Source Data [file 41467_2018_8157_MOESM4_ESM.zip › source data-14122018/Realtive_uptakes_wt-PGC1_M280I-PGC1-SupFig14/26 PPAR[399-406] AVKFNALE.png]

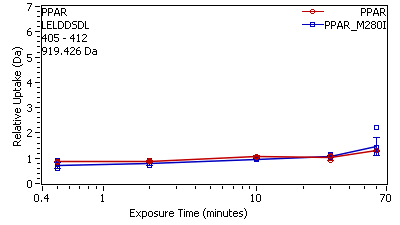

Supplement: Supplementary file 4 — Source Data [file 41467_2018_8157_MOESM4_ESM.zip › source data-14122018/Realtive_uptakes_wt-PGC1_M280I-PGC1-SupFig14/27 PPAR[405-412] LELDDSDL.png]

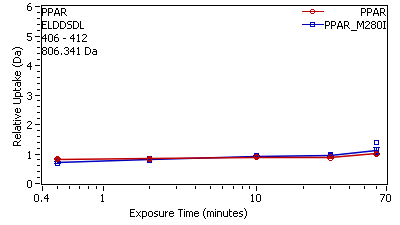

Supplement: Supplementary file 4 — Source Data [file 41467_2018_8157_MOESM4_ESM.zip › source data-14122018/Realtive_uptakes_wt-PGC1_M280I-PGC1-SupFig14/28 PPAR[406-412] ELDDSDL.png]

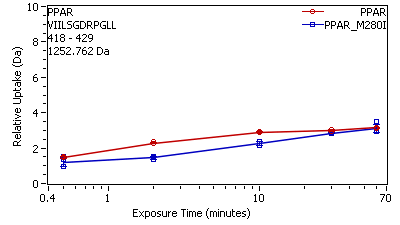

Supplement: Supplementary file 4 — Source Data [file 41467_2018_8157_MOESM4_ESM.zip › source data-14122018/Realtive_uptakes_wt-PGC1_M280I-PGC1-SupFig14/29 PPAR[418-429] VIILSGDRPGLL.png]

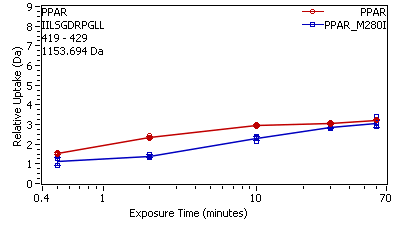

Supplement: Supplementary file 4 — Source Data [file 41467_2018_8157_MOESM4_ESM.zip › source data-14122018/Realtive_uptakes_wt-PGC1_M280I-PGC1-SupFig14/30 PPAR[419-429] IILSGDRPGLL.png]

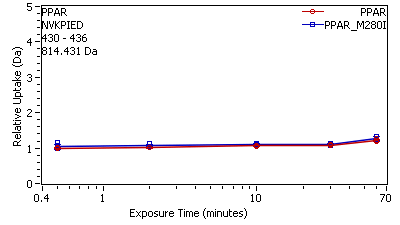

Supplement: Supplementary file 4 — Source Data [file 41467_2018_8157_MOESM4_ESM.zip › source data-14122018/Realtive_uptakes_wt-PGC1_M280I-PGC1-SupFig14/31 PPAR[430-436] NVKPIED.png]

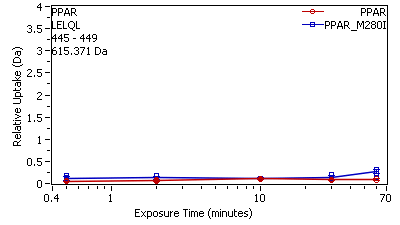

Supplement: Supplementary file 4 — Source Data [file 41467_2018_8157_MOESM4_ESM.zip › source data-14122018/Realtive_uptakes_wt-PGC1_M280I-PGC1-SupFig14/32 PPAR[445-449] LELQL.png]

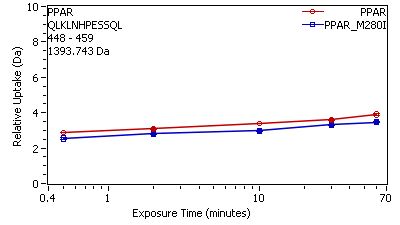

Supplement: Supplementary file 4 — Source Data [file 41467_2018_8157_MOESM4_ESM.zip › source data-14122018/Realtive_uptakes_wt-PGC1_M280I-PGC1-SupFig14/33 PPAR[448-459] QLKLNHPESSQL.png]

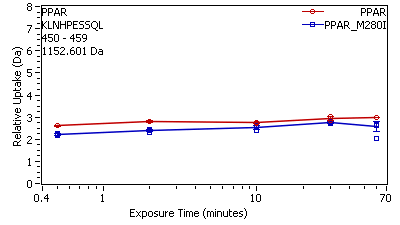

Supplement: Supplementary file 4 — Source Data [file 41467_2018_8157_MOESM4_ESM.zip › source data-14122018/Realtive_uptakes_wt-PGC1_M280I-PGC1-SupFig14/34 PPAR[450-459] KLNHPESSQL.png]

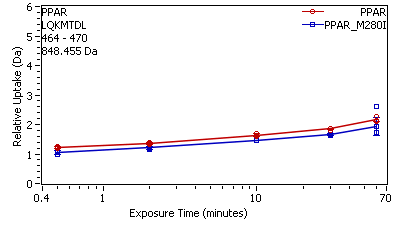

Supplement: Supplementary file 4 — Source Data [file 41467_2018_8157_MOESM4_ESM.zip › source data-14122018/Realtive_uptakes_wt-PGC1_M280I-PGC1-SupFig14/35 PPAR[464-470] LQKMTDL.png]

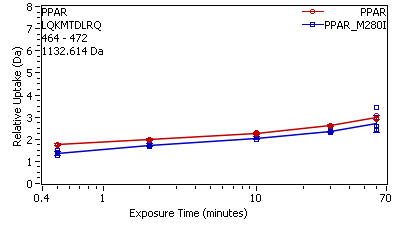

Supplement: Supplementary file 4 — Source Data [file 41467_2018_8157_MOESM4_ESM.zip › source data-14122018/Realtive_uptakes_wt-PGC1_M280I-PGC1-SupFig14/36 PPAR[464-472] LQKMTDLRQ.png]

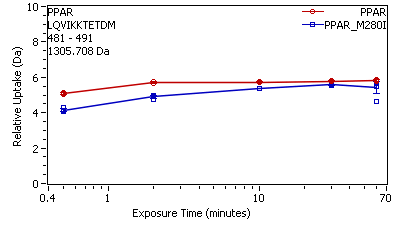

Supplement: Supplementary file 4 — Source Data [file 41467_2018_8157_MOESM4_ESM.zip › source data-14122018/Realtive_uptakes_wt-PGC1_M280I-PGC1-SupFig14/37 PPAR[481-491] LQVIKKTETDM.png]

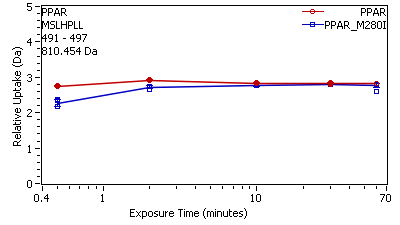

Supplement: Supplementary file 4 — Source Data [file 41467_2018_8157_MOESM4_ESM.zip › source data-14122018/Realtive_uptakes_wt-PGC1_M280I-PGC1-SupFig14/38 PPAR[491-497] MSLHPLL.png]

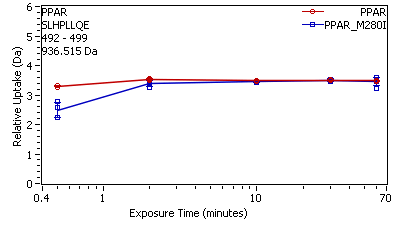

Supplement: Supplementary file 4 — Source Data [file 41467_2018_8157_MOESM4_ESM.zip › source data-14122018/Realtive_uptakes_wt-PGC1_M280I-PGC1-SupFig14/39 PPAR[492-499] SLHPLLQE.png]

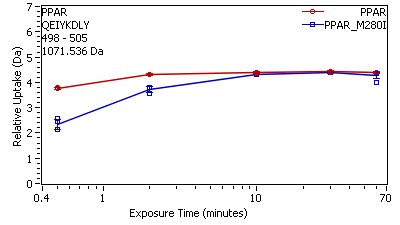

Supplement: Supplementary file 4 — Source Data [file 41467_2018_8157_MOESM4_ESM.zip › source data-14122018/Realtive_uptakes_wt-PGC1_M280I-PGC1-SupFig14/40 PPAR[498-505] QEIYKDLY.png]
